# Association of artificial sweeteners intake and risk of CKD: a prospective cohort study

**DOI:** 10.1016/j.jnha.2026.100917

**Published:** 2026-06-30

**Authors:** Jian Wang, Anwen Wang, Bo Liu, Yuanyuan Cao, Tao Sun, Xingyuan Zhang, Lijin Lin, Xuewei Huang, Weifang Liu, Wenyu Yang, Dongdong Tian, Fang Lei

**Affiliations:** a Department of Nephrology, Qichun County Renmin Hospital, Huanggang, China; b Medical Science Research Center, Zhongnan Hospital of Wuhan University, Wuhan, China; c Department of Cardiology, Renmin Hospital of Wuhan University, Wuhan, China; d School of Basic Medical Science, Wuhan University, Wuhan, China; e Department of Cardiology, The Third Xiangya Hospital, Central South University, Changsha, China; f State Key Laboratory of New Drug Discovery and Development for Major Diseases, Gannan Medical University, Ganzhou, China; g Gannan Innovation and Translational Medicine Research Institute, Gannan Medical University, Ganzhou, China; h Department of Pharmacy, Qichun County Renmin Hospital, Huanggang, China

**Keywords:** Artificial sweetener, Chronic kidney disease, Polygenic risk score, Interaction

## Abstract

**Background:**

Artificial sweeteners (AS) are widely used in foods and beverages as low-calorie alternative, but their safety remains controversial. Robust epidemiological evidence regarding the impact of AS on kidney health, especially the risk of chronic kidney disease (CKD) is currently lacking.

**Methods:**

The study included 156,000 participants from the UK Biobank cohort with a median follow-up of 13.3 years. The intake of AS from various sources was evaluated through a 24-h dietary recall. Incident CKD was defined as a newly diagnosed by the ICD codes and follow-up eGFR from study records and general practice data. Cox regression models were used to analyze the association between AS intakes and the risk of incident CKD, as well as the combined effects between genetic risk and AS intakes on incident CKD.

**Results:**

Compared to None-AS group, high-AS group had higher risk of new-onset CKD after fully adjusted, with HR 1.19 (95% CI:1.08−1.30). Participants with high AS intakes and high polygenic risk had the greatest risk of CKD (HR = 1.49, 95% CI:1.28−1.74). In substitution analysis, replacing sugar with an equivalent sweetness from AS resulted in an HR of 1.02 (95% CI: 1.01–1.04) for incident CKD.

**Conclusions:**

AS intake is associated with an increased risk of incident CKD, an association that exists across all levels of CKD genetic risk. Substitution analysis indicate no advantage to using AS instead of sugar to reduce the risk of CKD. These findings provide essential evidence for reassessing the role of AS intake contributes to the new-onset CKD.

## Graphical abstract

**Figure:**
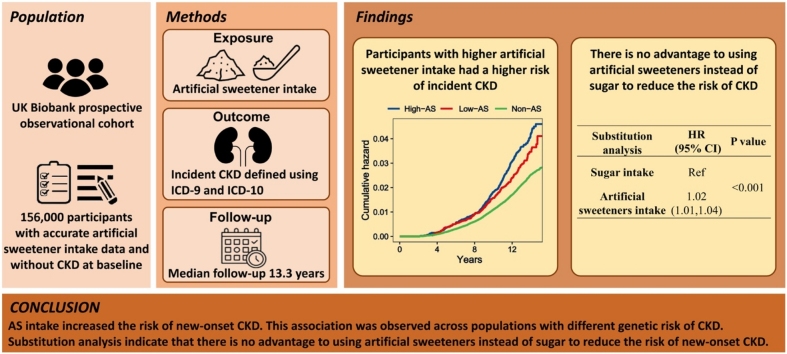

## 1 Introduction

Chronic kidney disease (CKD) is one of the major contributors to health burden worldwide [1]. The global incidence of CKD rose from 7.80 million cases in 1990 to 18.99 million cases in 2019 [2,3]. Facing such a rapid increase in the disease burden, a good understanding of risk factors is a critical step in formulating strategies to control the CKD pandemic. Many risk factors associated with CKD have been established, such as hyperglycemia, high blood pressure, overactivation of renin-angiotensin aldosterone system (RAAS) and dyslipidemia [4].

Recently, evidence suggests artificial sweetener beverages (ASB) may increase the risk of new-onset CKD [[5], [6], [7]]. However, the results from studies were not entirely consistent. In a community study, the participants who consumed diet soda had a higher risk of developing end-stage renal disease compared to non-consumers [8]. In the Nurses' Health Study, the participants who consumed ≥2 servings of artificially sweetened soda daily significantly increased their risk for estimated glomerular filtration rate (eGFR) decline [5]. A UK Biobank study indicated that reduced intake of ASB might lower the risk of CKD development [7]. While in a case-control study and Meta-analyses, the intake of artificially sweetened beverages was not significantly associated with CKD [6,[9], [10], [11]]. Study design, sample sizes, and population heterogeneity may cause such differences. It is crucial to acknowledge that the abovementioned studies explored the association between the consumption of ASB and CKD risk. In addition to artificial sweeteners (AS), these beverages contain other ingredients, such as phosphorus and dietary acid, which may also contribute to the development of CKD [[12], [13], [14]]. Furthermore, these studies overlooked the consumption of tabletop AS, like Canderel, which significantly contribute to artificial sweetener intake. The relationship between artificial sweetener consumption (excluding artificially sweetened beverages as a variable) and the risk of developing CKD requires investigation. Moreover, the potential influence of polygenic risk for CKD on the relationship between AS consumption and the risk of developing CKD is yet to be determined.

Therefore, we analyzed the association between dietary AS intake and incident CKD risk and the combined effects between polygenic risk and artificial sweetener intakes on incident CKD in a large-scale prospective UK Biobank cohort. We further conducted a substitution analysis to verify whether AS are a safe alternative to sugar for the risk of CKD incidence. Last, we conducted subgroup analysis to identify high-risk populations in which artificial sweetener intake is associated with a higher risk of incident CKD. These findings contribute to a better understanding of emerging risk factor on CKD and may inform future dietary guidelines and preventative measures.

## 2 Materials and methods

### 2.1 Data source and study population

The UK Biobank is a prospective cohort study that enrolled approximately 500,000 participants aged 40–69 from across the UK between 2006–2010 [[15], [16], [17]]. Enrollment involved participants visiting one of the 22 assessment centers, where baseline data on social demographics, physical assessments, health and lifestyle were collected through physical measurements and touch-screen questionnaires [18,19].

**Fig. 1:**
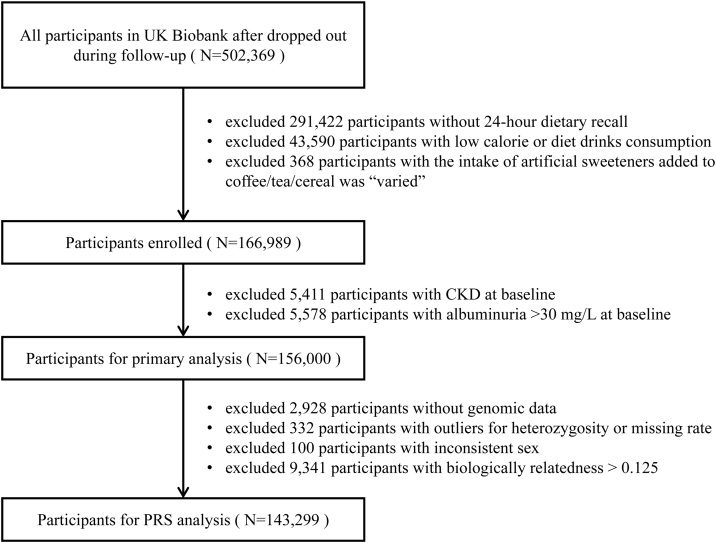
The flowchart shows the strategy of participant selection from the UK Biobank cohort. A schematic overview illustrating participants' enrollment and the exclusion and inclusion criteria.

In this study, we collected participants with 24 -h dietary recall of the previous day to assess the intake of AS. Fig. 1 illustrates the participant selection process. We excluded 291,422 participants without 24 -h dietary recall. All remaining participants consuming coffee, tea, and cereal, along with details on the use of tabletop AS. We further excluded 43,590 participants who consumed low-calorie or diet drinks. Low-calorie beverages may include naturally or artificially derived sweeteners and other additive components, and the exact dosage of AS can not be obtained. We excluded 368 participants whose the intake of AS added to cereal, tea or coffee was “varied” because we were unable to calculate their artificial sweetener consumption accurately. We excluded 5,411 participants with CKD at baseline. Finally, we excluded 5,578 participants with albuminuria >30 mg/L at baseline. The study included the remaining 156,000 participants.

In the genetic risk stratification analysis, we further excluded participants without genomic data (n = 2,928), Participants with outliers for missing rate and heterozygosity (n = 332), with inconsistent sex (n = 100) and the biologically relatedness greater than 0.125 (n = 9,341) were also excluded [20]. This threshold was applied to ensure the statistical independence of observations and to eliminate potential bias from second-degree relatives or closer in the polygenic risk score (PRS) analysis. Finally, there were 143,299 participants included in this analysis.

Ethical approval was granted by the North West Multicenter Research Ethical Committee, and participants gave written consent. This research was carried out under UK Biobank application number 77195.

### 2.2 Artificial sweetener intake

AS intake was evaluated through a 24-h dietary recall using the Oxford WebQ, a web-based dietary assessment tool available at www.ceu.ox.ac.uk/research/oxford- webq. This tool records the consumption of 206 consumed foods and 32 beverages types over the past 24 h [21]. UK Biobank participants completed the questionnnaire five times over five years, and the available data were used to compute the mean values. As previously explained, following a comprehensive review of all data fields, we included the following data to assess AS intake: "Intake of AS added to cereal," "Intake of AS added to tea," and "Intake of AS added to coffee" (Supplemental Table S1). Participants were asked the following question: "How many teaspoons/tablets of sweetener (e.g., Canderel) did you add to your coffee (per drink)? /add to your tea/infusion (per drink)? /add to your cereal or porridge (per bowl)?". Calculating AS intake requires precise information about coffee (cups/mugs), tea (cups/mugs), and cereal (bowls) consumption. We conducted additional screening of data fields: The total coffee consumption was determined by summing the daily cups from six mutually exclusive coffee types: "Instant coffee intake," "Filtered coffee intake," "Cappuccino intake," "Latte intake," "Espresso intake," and "Other coffee type." Total cereal consumption was similarly determined by aggregating the bowls from eight mutually exclusive cereal types: "Porridge intake," "Muesli intake," "Oat crunch intake," "Sweetened cereal intake," "Plain cereal intake," "Bran cereal intake," "Whole-wheat cereal intake," and "Other cereal intake." Ultimately, we calculate the AS consumption for each study participant [22].

Participants were divided into three groups according to the quantity of AS intake. Participants with no AS intake were referred to as the None-AS group. Individuals who consumed AS were divided into Low-AS (artificial sweetener intake <4 teaspoons per day) and High-AS groups (artificial sweetener intake ≥4 teaspoons per day) according to the median level (4.0 teaspoons per day) of AS intake.

### 2.3 Chronic kidney disease determination

The incident of CKD was determined by either the presence of diagnostic codes or eGFR below 60 mL/min/1.73m^2^, whichever came first during follow-up time [23,24]. The diagnostic codes indicated CKD in the International Classification of Diseases (ICD), specifically the Ninth Revision (ICD-9) and Tenth Revision (ICD-10). For ICD-9, the specific codes were 585 and 5859, while for ICD-10, the codes were I12.0, I13.1, I13.2, N18, N18.0, N18.1, N18.2, N18.3, N18.4, N18.5, N18.8, and N18.9 [25]. Follow-up serum creatinine was obtained from the UK Biobank and linked to GP records. eGFR were estimated using the 2021 CKD‐EPI creatinine equations [26,27].

### 2.4 Polygenic risk score for chronic kidney disease

The polygenic risk score (PRS) was constructed from the genome-wide association studies on kidney function involving one million individuals [28]. 251 SNPs were used to calculate the weighted PRS according to the formula: PRS_CKD_ = β_1_*SNP_1_ + β_2_*SNP_2_ + … + β_251_*SNP_251_. The selected SNPs information are reported in Supplemental Table S2. A comprehensive account of the genotyping platform and imputation strategy can be found in a prior publication [20,29]. Participants were subsequently grouped into low, intermediate and high genetic risk according to the tertiles of PRS_CKD_. The association between PRS_CKD_ stratification and the incidence of CKD was assessed using Cox regression models. We further analyze the combined effects between genetic risk and artificial sweetener intakes on incident CKD.

### 2.5 Covariates

We considered a variety of potential confounders. Data pertaining to lifestyle factors socio-demographics, habitual diet and medical history was obtained by administering touch screen questionnaires. The collection of anthropometric data involved conducting physical measurements and biochemical measurements.

Potential confounders include baseline age, sex (female, male), race (white, non-white), body-mass index (BMI) (underweight/normal weight (BMI < 25 kg/m^2^), overweight (25 ≤ BMI < 30 kg/m^2^), obese (BMI ≥ 30 kg/m^2^)), alcohol intake (not current, <3 times/week, ≥3 times/week), smoking (never, previous, current), Townsend deprivation index (categorized into quartiles), educational level (college/university degree, other qualification, no qualification), metabolic equivalents (METs) (categorized into quartiles), eGFR, number of glasses of coffee/tea/cereal per day, overall nutritional status (protein, energy, carbohydrate, total sugars, fat intake per day) and comorbidities (hypertension, dyslipidemia and diabetes).

### 2.6 Statistical analyses

Multivariate-adjusted Cox models were employed to calculate the hazard ratio (HR) to assess the relationship between AS intake and the risk of incident CKD with with time at recruitment as the start of follow-up. In Cox models, the None-AS group was set as the reference.

To minimize potential inferential bias and maximize statistical power, the MissForest algorithm was employed to impute missing data. MissForest is an iterative, non-parametric imputation technique based on random forests that does not require explicit assumptions regarding the distribution of the data. It functions by predicting missing values for each variable using a random forest model trained on the observed values of all other variables. This method is robust for capturing complex, non-linear interactions within the dataset [[30], [31], [32]]. Supplemental Table S3 provides detailed information regarding missing covariates of participants.

The Schoenfeld residuals method confirmed that the Cox models met the proportional hazards assumption without any violations. Model 1 adjusted age, sex and race. In model 2, BMI, eGFR, smoking, drinking, education level, Townsend deprivation index, and total metabolic equivalent weekly task minutes were additionally adjusted. Model 3 further adjusted the coffee, tea, cereal, protein, carbohydrate, energy, total sugars, fat intakes, and comorbidities (history of hypertension, diabetes and dyslipidemia). To evaluate the potential interaction effects, the likelihood ratio test was used to compare the multivariate Cox models with and without interaction terms.

Individuals with multiple serum creatinine measurement during follow-up time were used to calculate eGFR slope. Multivariate-adjusted linear regression (LR) models were employed to calculate the beta and 95%CI to assess the association between artificial sweetener intake and eGFR slope. We also used the LR model to analyze the association between artificial sweetener intake and CKD onset time among participants who developed CKD during follow-up.

A substitution analysis was employed to evaluate the theoretical effect of replacing sugar intake with an equivalent sweetness from artificial sweeteners [33]. This model estimated the hazard ratio HR for a 1 teaspoon/day increased intake of tabletop artificial sweeteners and a concomitantly decreased sugar intake of equal sweetness (2 teaspoons ≈ 9 g/day). This approach follows established food substitution models in nutritional epidemiology to compare the relative risks of different dietary components while controlling for total energy and sweetness [34,35].

All statistical analyses were performed using R version 4.3.0. Group comparisons employed ANOVA for normally distributed variables, the Mann–Whitney U test for non-parametric variables, and either Fisher’s exact test or the chi-squared test for categorical variables.

### 2.7 Sensitivity analyses

We performed multiple sensitivity analyses to evaluate the robustness of our results. First, We incorporated propensity score matching (PSM) to match participants in High-AS group and participants in None-AS group for addressing confounding and improving balance between the exposure groups. We match participants in High-AS group and participants in Low-AS group with the matching ratio 1:4 using PSM with a caliper size of 0.05. Standardized differences were estimated post-matching to assess covariate balance.Balancing was deemed qualified only if the absolute value was less than 0.1 [31]. A Cox model was used to assess the association between the AS intake and CKD incident risk in the matched cohorts.

In sensitivity analysis II, we further adjusted vegetable, red/processed meat and fruit intake. Considering the potential impact of carbonated beverages on CKD risk, we adjusted for the intake of fizzy drink in sensitivity analysis III.

In sensitivity analysis IV, a mixed-effects Cox model was used to adjust the effect of centers by considering the UK Biobank assessment center sites as random effects [36,37]. To avoid underestimating the incidence of CKD that can result from censoring for death, sensitivity analysis V was performed using Fine and Gray’s competing risk regression, adjusted for the competing risk of death [38,39].

In sensitivity analysis VI-VIII, to further avoid reverse causality, we sequentially excluded participants who developed CKD within initial two, three, and five years of follow-up, and then assessed the relationship between AS intake and the CKD risk.

## 3 Results

### 3.1 Descriptive population characteristics

Table 1 presents the baseline characteristics of the study participants. The final analysis included 156,000 adults. The mean age of this population was 56.8 (standard deviation [SD] 7.9) years. 85,020 (54.5%) participants were female. The mean eGFR was 95.5 (SD 11.4) ml/min/1.73 m^2^ and the mean BMI was 26.4 (SD 4.3) kg/m^2^. The mean artificial sweetener intake was 1.8 (SD 1.0) teaspoons per day in the Low-AS group and 7.3 (SD 3.5) teaspoons per day in High-AS group. Compared with the lower artificial sweetener intake population, higher artificial sweetener intake participants tended to be older, less educated, and more materially deprived. The proportion of current smokers was slightly higher in High-AS group than None-AS group, but None-AS group had a higher proportion of alcohol consumption. The proportions with histories of hypertension, dyslipidemia and diabetes were higher in the High-AS group than None-AS group and Low-AS group (Table 1). Additionally, in comparison with the entire UK Biobank cohort, the participants included in our study had similar age (mean (SD): 56.8 (7.9) years vs. 57.0 (8.1) years), lower rate of current smoking (7.9% vs. 10.6%), lower townsend deprivation index and a lower percentage of comorbidities (Supplemental Table S4).

**Table 1:** Characteristics of participants in the longitudinal cohort.

		Artificial sweetener intake			
Characteristics	Overall N = 156,000	None-AS N = 140,196	Low-AS N = 7,565	High-AS N = 8,239	P value^a^
Age (year, mean (SD))	56.8 (7.9)	56.6 (7.9)	57.9 (7.8)	58.4 (7.5)	<0.001
Gender, Female, n (%)	85020 (54.5)	76417 (54.5)	4469 (59.1)	4134 (50.2)	<0.001
Race, n(%)					<0.001
White	148843 (95.8)	133803 (95.8)	7116 (94.3)	7924 (96.6)	
Non-white	6565 (4.2)	5856 (4.2)	427 (5.7)	282 (3.4)	
Cigarette smoking, n (%)					<0.001
Never	88975 (57.2)	81708 (58.4)	3864 (51.3)	3403 (41.4)	
Previous	54316 (34.9)	47502 (34.0)	3107 (41.2)	3707 (45.1)	
Current	12293 (7.9)	10621 (7.6)	568 (7.5)	1104 (13.4)	
Alcohol consumption, n (%)					<0.001
Not current	9267 (5.9)	8051 (5.7)	510 (6.8)	706 (8.6)	
Two or fewer times a week	69575 (44.6)	61573 (44.0)	3742 (49.5)	4260 (51.7)	
Three or more times a week	77032 (49.4)	70457 (50.3)	3303 (43.7)	3272 (39.7)	
Education level, n (%)					<0.001
No qualification	12846 (8.3)	10713 (7.7)	864 (11.5)	1269 (15.5)	
Any other qualification	73730 (47.5)	64914 (46.5)	4131 (54.9)	4685 (57.2)	
Degree or above	68677 (44.2)	63909 (45.8)	2529 (33.6)	2239 (27.3)	
Townsend deprivation index, (mean (SD))	−1.6 (2.9)	−1.6 (2.9)	−1.6 (2.9)	−1.4 (3.0)	<0.001
BMI (kg/m^2^, mean (SD))	26.4 (4.3)	26.3 (4.3)	27.7 (4.6)	28.2 (4.6)	<0.001
BMI category (kg/m2), n (%)					<0.001
<25	64010 (41.1)	59800 (42.8)	2202 (29.2)	2008 (24.5)	
25–30	64340 (41.4)	57055 (40.8)	3420 (45.3)	3865 (47.1)	
≥30	27222 (17.5)	22965 (16.4)	1920 (25.5)	2337 (28.5)	
eGFR, (ml/min/1.73 m^2^, mean (SD))	95.5 (11.4)	95.6 (11.3)	94.6 (11.4)	93.9 (11.5)	<0.001
Artificial sweetener intake (teaspoon/day, mean (SD))	0.5 (1.9)	0.0 (0.0)	1.8 (1.0)	7.3 (3.5)	<0.001
Total sugars intake (g/day, mean (SD))	125.5 (49.3)	125.7 (49.2)	123.0 (49.3)	125.1 (52.2)	<0.001
Diabetes history, n (%)	9126 (5.9)	7307 (5.2)	794 (10.5)	1025 (12.4)	<0.001
Hypertension history, n (%)	71982 (46.1)	63678 (45.4)	3876 (51.2)	4428 (53.7)	<0.001
Dyslipidemia history, n (%)	68223 (43.7)	60952 (43.5)	3447 (45.6)	3824 (46.4)	<0.001

Abbreviation: eGFR, estimated glomerular filtration rate; BMI, body-mass index; SD, standard deviation.

Townsend deprivation index composite area-level measure of deprivation based on unemployment, non-car ownership, non-home ownership, and household overcrowding; a higher score indicates higher deprivation.

a Group comparisons were executed using analysis of variance (ANOVA) for normally distributed variables, the Mann–Whitney U test for non-parametric variables, and either Fisher’s exact test or the chi-squared test for categorical variables.

### 3.2 Associations between artificial sweetener intake and the incidence of chronic kidney disease

Over a median follow-up period of 13.3 years (interquartile range[IQR], 12.7–14.1), 6,022 participants developed CKD and were diagnosed. The incidence rate (IR) of CKD in None-AS group, Low-AS group, and High-AS group were 2.75 (95% CI: 2.68–2.83), 3.85 (95% CI:3.46−4.23) and 4.89 (95% CI:4.47−5.30) per 1000 person- years, respectively. Participants in the Low-AS and High-AS groups exhibited higher risks of developing CKD during follow-up compared to the None-AS group, with crude HRs of 1.40 (95% CI, 1.26–1.55, p < 0.001) and 1.78 (95% CI, 1.63–1.95, p < 0.001), respectively. Even after comprehensive adjustment for potential confounders, the High-AS group maintained a significant association, with an HR of 1.19 (95% CI, 1.08–1.30, p < 0.001) (Table 2). We also estimated the impact of artificial sweetener intake, as a continuous variable, on CKD incidence, and it was still significantly associated with incident CKD risk, with a multivariate-adjusted HR (per 1 SD increase in artificial sweetener intake) of 1.04 (95% CI,1.02–1.06, p < 0.001) (Supplemental Table S5). We further analyzed the association between AS intake and eGFR slope in participants with follow-up serum creatinine measurements. After fully adjusted, compared with participants in the None-AS group, those in the High-AS group had a steeper eGFR slope, with fully adjusted beta -0.34(95% CI:-0.65,-0.03, p = 0.03) (Supplemental Table S6). Additionally, we analyzed the relationship between artificial sweetener intake and time to incident CKD among participants who developed CKD during the follow-up. We found no statistically significant association between artificial sweetener intake and the time to CKD onset (Supplemental Table S7).

**Table 2:** Association between artificial sweetener intake group and incidence of CKD in the longitudinal cohort.

		IR (95% CI)	Crude	Model l^a^	Model 2^b^	Model 3^c^
AS intake	Cases/Total	1000 Person-years	HR (95% CI); P	HR (95% CI); P	HR (95% CI); P	HR (95% CI); P
None-AS	5113/140196	2.75 (2.68,2.83)	Ref	Ref	Ref	Ref
Low-AS	383/7565	3.85 (3.46,4.23)	1.40 (1.26,1.55)	1.24 (1.12,1.38)	1.09 (0.99,1.21)	1.05 (0.94,1.16)
			<0.001	<0.001	0.09	0.39
High-AS	526/8239	4.89 (4.47,5.30)	1.78 (1.63,1.95)	1.52 (1.39,1.67)	1.24 (1.13,1.36)	1.19 (1.08,1.30)
			<0.001	<0.001	<0.001	<0.001

Abbreviation: CKD, chronic kidney disease; AS, artificial sweetener; eGFR, estimated glomerular filtration rate; BMI, body-mass index; IR, incidence rate; HR, hazard ratio; CI, confidence interval; Ref, reference.

a Model 1 the adjustment factors included age, sex and race.

b Model 2 the adjustment factors included age, sex, race, BMI, eGFR, smoking, drinking, education level, townsend deprivation index and total MET minutes per week.

c Model 3 the adjustment factors included age, sex, race, BMI, eGFR, smoking, drinking, education level, townsend deprivation index, total MET minutes per week, coffee intake, tea intake, cereal intake, protein intake, carbohydrate intake, energy intake, total sugars intake, fat intake and comorbidities (history of hypertension, diabetes and dyslipidemia).

To further analyze whether artificial sweeteners are a safe substitute for sugar in relation to CKD risk, we conducted a substitution analysis. In the substitution analysis, replacing 9 g of sugar with an equivalent sweetness from 1 teaspoon of artificial sweeteners was associated with a small but significant increase in CKD risk (fully adjusted HR = 1.02; 95% CI: 1.01–1.04) (Supplemental Table S8). These findings suggest that replacing sugar with artificial sweeteners may not offer a protective benefit against the development of new-onset CKD.

**Fig. 2:**
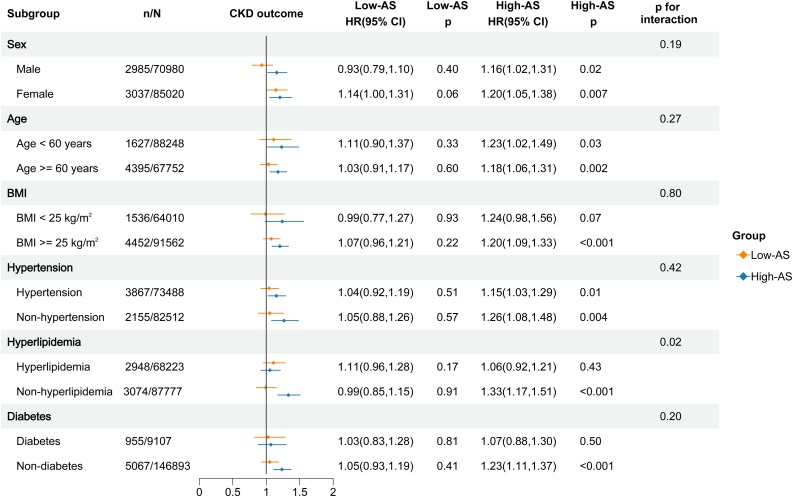
Association between artificial sweetener intake and the incidence of chronic kidney disease in the cohort subgroups. The adjustment factors included age, sex, ethnic, BMI, eGFR, smoking, drinking, education level, townsend deprivation index, total MET minutes per week, coffee intake, tea intake, cereal intake, protein intake, carbohydrate intake, energy intake, total sugars intake, fat intake and comorbidities (history of hypertension, history of diabetes and history of dyslipidemia). Abbreviation: CKD, chronic kidney disease; AS, artificial sweetener; HR, hazard ratio; CI, confidence interval.

The subgroup analysis revealed that compared with None-AS group, participants in High-AS group exhibited a significantly higher risk of CKD incidence in male and female subgroup, all age subgroup, BMI ≥ 25 kg/m^2^ subgroup, non-dyslipidemia subgroup, non-diabetes subgroup and both hypertension and non-hypertension subgroups (Fig. 2). A significant interaction effect on the risk of incident CKD was observed between artificial sweetener intake levels and baseline dyslipidemia status (P_interaction_ = 0.02). This interaction suggests that the association between high AS intake and CKD risk may be more pronounced in participants without dyslipidemia.

In summary, participants with high-dose artificial sweetener intake had a higher CKD incident risk, particularly among females, younger participants and those without metabolic disease.

### 3.3 Combined effects between genetic risk and artificial sweetener intake on incidence of chronic kidney disease

For genetics risk analyses, we found a significant association between CKD-PRS and the risk of incident CKD. Participants with high polygenic risk scores had approximately and 35% (95% CI 27%–45%, p < 0.001) higher risk of incident CKD compared to those with low polygenic risk scores (Supplemental Table S9).

We estimated the joint effects of AS intake and CKD-PRS on the risk of incident CKD. It was found that participants taking high artificial sweeteners and with high polygenic risk had a higher risk of incident CKD (HR = 1.49, 95% CI: 1.28–1.74, p < 0.001) than those taking low artificial sweeteners and with low polygenic risk (Table 3). Besides, the interaction analysis revealed that there was no significant interaction between AS intake and the polygenic risk score (P_interaction_ = 0.23), indicating the associations between AS intake and CKD incident did not substantially vary according to polygenic risk (Table 3).

**Table 3:** Risk of incident CKD according to genetic risk and artificial sweetener intake in 143,299 UK Biobank participants.

		IR (95%CI)	Crude		Model 1^a^		Model 2^b^		Model 3^c^		
	Cases/Total	1000 Person-years	HR (95%CI)	P value	HR (95%CI)	P value	HR (95%CI)	P value	HR (95%CI)	P value	P for interaction
Low-PRS											0.23
None-AS	1185/42825	2.08 (1.96,2.20)	Ref	–	Ref	–	Ref	–	Ref	–	
Low-AS	83/2377	2.63 (2.07,3.20)	1.26 (1.01,1.58)	0.04	1.12 (0.90,1.40)	0.30	1.01 (0.81,1.26)	0.96	0.96 (0.77,1.20)	0.70	
High-AS	142/2562	4.22 (3.53,4.91)	2.03 (1.71,2.42)	<0.001	1.73 (1.45,2.05)	<0.001	1.43 (1.20,1.70)	<0.001	1.38 (1.16,1.65)	<0.001	
Intermediate-PRS											
None-AS	1468/42930	2.58 (2.44,2.71)	1.24 (1.15,1.34)	<0.001	1.25 (1.15,1.34)	<0.001	1.11 (1.02,1.19)	0.01	1.11 (1.03,1.20)	0.009	
Low-AS	116/2324	3.78 (3.09,4.47)	1.82 (1.50,2.20)	<0.001	1.60 (1.32,1.93)	<0.001	1.25 (1.03,1.52)	0.02	1.20 (0.99,1.45)	0.07	
High-AS	142/2505	4.32 (3.61,5.03)	2.08 (1.75,2.48)	<0.001	1.79 (1.51,2.13)	<0.001	1.30 (1.09,1.55)	0.003	1.24 (1.04,1.48)	0.02	
High-PRS											
None-AS	2048/43046	3.61 (3.45,3.76)	1.73 (1.61,1.86)	<0.001	1.75 (1.63,1.88)	<0.001	1.37 (1.27,1.47)	<0.001	1.37 (1.28,1.48)	<0.001	
Low-AS	148/2242	5.06 (4.25,5.88)	2.44 (2.05,2.89)	<0.001	2.19 (1.85,2.60)	<0.001	1.48 (1.25,1.75)	<0.001	1.44 (1.22,1.71)	<0.001	
High-AS	191/2488	5.91 (5.07,6.75)	2.85 (2.44,3.32)	<0.001	2.49 (2.14,2.90)	<0.001	1.55 (1.33,1.81)	<0.001	1.49 (1.28,1.74)	<0.001	

Abbreviation: CKD, chronic kidney disease; PRS, polygenic risk score; AS, artificial sweetener; eGFR, estimated glomerular filtration rate; BMI, body-mass index; IR, incidence rate; HR, hazard ratio; CI, confidence interval; Ref, reference.

a Model 1 the adjustment factors included age, sex and race.

b Model 2 the adjustment factors included age, sex, race, BMI, eGFR, smoking, drinking, education level, townsend deprivation index and total MET minutes per week.

c Model 3 the adjustment factors included age, sex, race, BMI, eGFR, smoking, drinking, education level, townsend deprivation index, total MET minutes per week, coffee intake, tea intake, cereal intake, protein intake, carbohydrate intake, energy intake, total sugars intake, fat intake and comorbidities (history of hypertension, history of diabetes and history of dyslipidemia).

### 3.4 Sensitivity analyses

Eight sensitivity analyses were conducted to assess the robustness of the the longitudinal study results.

In sensitivity analysis I, we use propensity score matching to address confounding and improve balance between the exposure groups After PSM, 32,640 participants in None-AS group and 8,160 participants in High-AS group were included in further analysis. The baseline information after PSM is presented in Supplemental Table S10 and balance was achieved among all confounding factors. By applying Cox model, the results consistently and significantly demonstrated a higher risk of new-onset CKD in the participants from the High-AS group (HR, 1.09; 95% CI, 1.04–1.14; p < 0.001) compared to those from the None-AS group (Supplemental Table S11).

In sensitivity analysis II, considering the potential effects of diet on CKD, we further adjusted the intake of fruit, vegetables and red/processed meat, respectively in the Cox models. AS intake were repeatedly associated with CKD incident (Supplemental Table S12). In sensitivity analysis III, we adjusted for the intake of fizzy drinks, and the association between AS intake and CKD risk remained significant (Supplemental Table S12).

In sensitivity analysis IV, we corrected the UK Biobank assessment center sites as random effects using a mixed-effects Cox model. A positive association of AS intake with the incidence of CKD exists (Supplemental Table S12).

In sensitivity analysis V, we performed competing risk analyses with death as a competing risk of the incidence of CKD. The analysis revealed that increased AS intake were still associated with increased risk for CKD (Supplemental Table S12).

In sensitivity analyses VI-VIII, the association of AS intake and the risk of new-onset CKD remains significant regardless of whether participants who developed CKD within 2 years, 3 years, or 5 years are excluded (Supplemental Table S13).

## 4 Discussion

This extensive cohort study identified a positive correlation between higher intake of AS and the risk of CKD incident. The association between high AS intake and incident CKD was consistent across various levels of polygenic risk. This observation suggests that the relationship between AS intake and CKD risk does not appear to be significantly modified by genetic predisposition. We also observed that AS are not a safe substitute for sugar in terms of CKD risk. These findings provide valuable insights for evidence of the association between AS intake and the risk of incident CKD and suggest no benefit from substituting AS for added sugar on CKD outcomes.

Currently, there is a lack of observational studies on the association between artificial sweeteners and the risk of incident CKD, making it hard to directly compare the results with other studies. However, several studies have used AS beverage consumption as a proxy for comparison study and meta-analysis [[5], [6], [7]]. The results and conclusions of these studies remain controversial. A cohort study of 3,318 female nurses found that participants with daily consumption of ≥2 servings of artificially sweetened soda per day significantly increases the risk of eGFR decline [5]. Another community study of 15,368 participants in the US also showed that diet soda intake was associated with the risk of incident end-stage renal disease during 23 years of follow-up [8]. A UK Biobank cohort study suggest that lower consumption of ASB may reduce the risk of developing CKD [7]. These are consistent with our findings. However, a case-control study of 465 patients with CKD and 467 community controls found that artificially sweetened colas intakes were not significantly associated with the prevalence of CKD [10]. This may be due to the small sample size. Meta-analyses performed by Lo Wei-Cheng et al. showed a positive association between the intake of ASB and the risk of incident CKD but did not reach statistical significance [9]. Although these studies bring interesting evidence, none can confirm whether the effects are due to artificial sweeteners or soda ingredients. In addition, the potentially strong reverse causality bias of design and unmeasured confounders limits the interpretability of these studies. Therefore, our study based on a prospective cohort with the largest sample size to date, adequately controlled for the potential confounding factors in low-calorie beverages. We found that the inkake of artificial sweeteners (not agent variable) were significantly associated with new-onset CKD, which is essential for establishing more authoritative knowledge about the effects of AS intake on kidney health.

There are potential mechanisms explaining the association between AS intake and the risk of CKD. Platelet aggregation, oxidative stress, systemic inflammation and metabolic disorders may be the potential mechanisms of the increased risk of CKD caused by artificial sweeteners [[40], [41], [42], [43], [44], [45]]. Elevated erythritol levels enhance platelet reactivity and thrombosis risk by promoting intracellular calcium release in platelets, potentially causing renal thrombosis and subsequent renal injury [41]. Artificial sweeteners can cause oxidative stress, resulting in increased reactive oxygen species (ROS) production, which in turn promotes tubular injury and apoptosis [46,47]. By altering the structure of the gut microbiota, Saccharin and sucralose consumption lead to systemic inflammation which directly contributes to CKD risk [40]. Multiple studies have found that artificial sweeteners increased the risk of metabolic syndrome, which is an important risk factor for CKD [[48], [49], [50], [51]].

In conclusion, our findings indicate that higher AS intake was associated with an heightened risk of incident CKD. Artificial sweeteners are extensively utilized as food additives and present in various products, including beverages, condiments, bread, cereals, dairy products, and other foods [52,53]. Our findings suggest that artificial sweeteners cannot be a safe alternative to sugar in food and may increase the risk of incident CKD. Our findings provide important insights for health institutions to reassess the risks of artificial sweeteners and also give the direction for investigating the mechanism by which artificial sweetener intake contributes to the onset of CKD. Additionally, further well-designed, large-scale prospective studies are required to confirm these findings.

This study has several important strengths. First, this study evaluated the relationship between artificial sweeteners and the risk of incident CKD from the intake of artificial sweeteners rather than low-calorie beverages based on a large prospective (N = 156,000) cohort. Second, the study accurately assesses individual-level exposure to artificial sweetener intake using repeated 24 -h dietary records. Most previous studies used ASB as a proxy to estimate AS intake. Last, this is the first study to assess the interaction effect of artificial sweeteners and genetics and non-genetic factors in the development of CKD to guide the prevention and control of high-risk populations.

However, several limitations in this study should be acknowledged. First, the study is an observational study, and the inherent limitations of selection bias cannot be excluded completely, even if extensive sensitivity analyses were used to demonstrate the robustness of the conclusions. Second, the minimum age of the study population was 40 years old at baseline, so generalization of the conclusion to the younger population requires caution. Third, measurement error in dietary intake cannot be eliminated entirely, even by collecting multiple 24-h questionnaires [54]. Fourthly, although we defined CKD using a composite standard of eGFR and ICD codes, there remains limited specificity in the CKD outcome due to the restricted availability of follow-up creatinine data [25]. Last, the different kinds of artificial sweeteners could not be distinguished in this study.

## 5 Conclusions

In large UK Biobank cohort, AS intake increased the risk of new-onset CKD. This association was observed across populations with different genetic risk of CKD. Substitution analysis indicate that there is no advantage to using artificial sweeteners instead of sugar to reduce the risk of new-onset CKD. These findings provide essential evidence for reassessing the risks of artificial sweeteners intake contributes to the new-onset CKD.

## Authorship contribution statement

Jian Wang: Data curation, Formal analysis, Writing-original draft. Anwen Wang: Data curation, Formal analysis, Writing-original draft. Bo Liu: Data curation. Yuanyuan Cao: Data curation. Tao Sun: Data curation. Xingyuan Zhang: Formal analysis. Lijin Lin: Formal analysis. Xuewei Huang: Writing-review & editing. Weifang Liu: Writing-review & editing. Wenyu Yang: Writing-review & editing. Dongdong Tian: Conceptualization, Supervision & Writing-review & editing. Fang Lei: Conceptualization, Funding acquisition, Project administration, Supervision, Writing-review & editing.

## Ethical statement

The study obtained ethical approval from the North West Multicenter Research Ethical Committee, and all participants provided written consent. This study was conducted under the UK Biobank application number 77195.

## Declaration of Generative AI and AI-assisted technologies in the writing process

No Generative AI or AI-assisted technologies were used in the scientific writing and in figures, images and artwork.

## Funding

This study was funded by the Medical Sci-Tech Innovation Platform of Zhongnan Hospital, Wuhan University (PTXM2025017) and the Hubei Provincial Natural Science Foundation of China (2024AFB887).

## Data availability

The data that support the findings of this study are available on request from the corresponding author. The UK Biobank data are available for approved researchers through the UK Biobank data-access protocol.

## Declaration of competing interest

The authors declare that they have no known competing financial interests or personal relationships that could have appeared to influence the work reported in this paper.
