# Corrigendum to “Association of artificial sweeteners intake and risk of CKD: a prospective cohort study” [J Nutr Health Aging, 30 (2026) 100917]

**DOI:** 10.1016/j.jnha.2026.100926

**Published:** 2026-07-23

**Authors:** Jian Wang, Anwen Wang, Bo Liu, Yuanyuan Cao, Tao Sun, Xingyuan Zhang, Lijin Lin, Xuewei Huang, Weifang Liu, Wenyu Yang, Dongdong Tian, Fang Lei

**Affiliations:** a Department of Nephrology, Qichun County Renmin Hospital, Huanggang, China; b Medical Science Research Center, Zhongnan Hospital of Wuhan University, Wuhan, China; c Department of Cardiology, Renmin Hospital of Wuhan University, Wuhan, China; d School of Basic Medical Science, Wuhan University, Wuhan, China; e Department of Cardiology, The Third Xiangya Hospital, Central South University, Changsha, China; f State Key Laboratory of New Drug Discovery and Development for Major Diseases, Gannan Medical University, Ganzhou, China; g Gannan Innovation and Translational Medicine Research Institute, Gannan Medical University, Ganzhou, China; h Department of Pharmacy, Qichun County Renmin Hospital, Huanggang, China

After publication, it came to the authors’ attention that references [42] and [45] were mistakenly cited in the Discussion section of their article, in the following sentence: "Platelet aggregation, oxidative stress, systemic inflammation and metabolic disorders may be the potential mechanisms of the increased risk of CKD caused by artificial sweeteners [40–45]."

Upon rechecking the cited literature, they acknowledge that references [42] and [45] were not appropriate to support this statement and should be removed from this citation group. Accordingly, the corrected sentence should read: "Platelet aggregation, oxidative stress, systemic inflammation and metabolic disorders may be the potential mechanisms of the increased risk of CKD caused by artificial sweeteners [40,41,43,44]."

The authors apologize for this error, which does not affect the study design, statistical analyses, results, or conclusions of the article, and for any inconvenience it may have caused.

