# Supplementary material for: Association of artificial sweeteners intake and risk of CKD: a prospective cohort study
Source: J Nutr Health Aging. 2026 Jun 30;30(8):100917. doi: 10.1016/j.jnha.2026.100917 (PMC13331974; doi:10.1016/j.jnha.2026.100917)
Supplement: Supplementary file 1 [file mmc1.docx]

**Association of artificial sweeteners intake and risk of chronic kidney disease: A prospective cohort study**

**Supplementary materials**

**Supplemental Table 1.** Details of items relevant to the calculation of artificial sweetener intake in dietary questionnaire based on a 24-hour dietary recall.

**Supplemental Table 2.** Components of the polygenic risk score of CKD.

**Supplemental Table 3.** The percentages of participants with missing covariates.

**Supplemental Table 4.** Characteristics of participants in this study and all participants in UK Biobank.

**Supplemental Table 5.** Association between the per SD increase of artificial sweetener intake and incidence of CKD in the longitudinal cohort.

**Supplemental Table 6.** Association between artificial sweetener intake and eGFR slope in participants with follow-up serum creatinine measurement.

**Supplemental Table 7.** Association between artificial sweeteners intake and time to incident CKD in the subcohort that included participants developed CKD with follow-up.

**Supplemental Table 8.** Substitution analysis of the association between risk of new-onset CKD and artificial sweeteners to replace sugar.

**Supplemental Table 9.** Associations of CKD genetic risk score with the risk of incident CKD in the longitudinal cohort.

**Supplemental Table 10.** Characteristics of all participants in the longitudinal cohort after PSM.

**Supplemental Table 11.** Sensitivity analysis I of the association between artificial sweetener intake and the incidence of CKD after PSM.

**Supplemental Table 12.** Sensitivity analysis II-V of the association between artificial sweetener intake and the incidence of CKD.

**Supplemental Table 13.** Sensitivity analysis VI-VIII of the association between artificial sweetener intake and the incidence of CKD by excluding events that occurred within the first two/three/five years of follow-up.

**Supplemental Table 1. Details of items relevant to the calculation of artificial sweetener intake in dietary questionnaire based on a 24-hour dietary recall.**

| **Questions Field** | **Note** | **Field ID** | **Code** |
| --- | --- | --- | --- |
| **All items of artificial sweetener** | | | |
| Intake of artificial sweetener added to coffee | How many teaspoons/tablets of sweetener (e.g. Canderel) did you add to your coffee (per drink)? | 100380 | 100002 |
| Intake of artificial sweetener added to tea | How many teaspoons/tablets of sweetener (e.g. Canderel) did you add to your tea/infusion (per drink)? | 100500 |  |
| Intake of artificial sweetener added to cereal | How much sweetener (e.g. Canderel) did you add to your cereal or porridge (per bowl)? | 100910 |  |
| **Items of coffee comsumption** | | | |
| Instant coffee intake | How many cups/mugs of instant coffee did you drink yesterday? | 100250 | 100006 |
| Filtered coffee intake | How many cups/mugs of filter/americano/cafetiere coffee did you drink yesterday? | 100270 |  |
| Cappuccino intake | How many cups/mugs of cappuccino did you drink yesterday? | 100290 |  |
| Latte intake | How many cups/mugs of latte did you drink yesterday? | 100300 |  |
| Espresso intake | How many cups/mugs of espresso did you drink yesterday? | 100310 |  |
| Other coffee type | How many cups/mugs of other coffee drinks did you drink yesterday? | 100330 |  |
| **Items of tea comsumption** | | | |
| Standard tea intake | How many cups/mugs of standard tea (e.g. Tetley, PG Tips, Assam, Darjeeling) did you drink yesterday? | 100400 | 100007 |
| Rooibos tea intake | How many cups/mugs of rooibos/redbush tea (e.g. Tick Tock) did you drink yesterday? | 100410 |  |
| Green tea intake | How many cups/mugs of green tea did you drink yesterday? | 100420 |  |
| Herbal tea intake | How many cups/mugs of herbal or fruit tea (infusion) did you drink yesterday? | 100430 |  |
| Other tea intake | How many cups/mugs of other tea or infusion did you drink yesterday? | 100440 |  |
| **Items of cereal comsumption** | | | |
| Porridge intake | How many bowls of porridge, hot oat cereal (e.g. Ready Brek)? | 100770 | 100001 |
| Muesli intake | How many bowls of muesli? | 100800 |  |
| Oat crunch intake | How many bowls of sweetened oat crunch type cereal (e.g. Jordans Country Crisp, chocolate nut clusters)? | 100810 |  |
| Sweetened cereal intake | How many bowls of other sweetened cereals (e.g. Ricicles, Honey Nut Cornflakes, Coco Pops)? | 100820 |  |
| Plain cereal intake | How many bowls of plain cereals (e.g. Cornflakes, Rice Krispies, Special K)? | 100830 |  |
| Bran cereal intake | How many bowls of bran cereals (e.g. bran flakes, All Bran)? | 100840 |  |
| Whole-wheat cereal intake | How many bowls of whole-wheat cereals (e.g. Weetabix, Shredded Wheat, Shreddies)? | 100850 |  |
| Other cereal intake | How many bowls of other cereal? | 100860 |  |

**Supplemental Table 2. Components of the polygenic risk score of CKD.**

| **SNP** | **CHR** | **BP** | **Effect allele** | **Beta** | **EAF** | **P value** | **Standard Error** |
| --- | --- | --- | --- | --- | --- | --- | --- |
| rs74748843 | 1 | 10730910 | T | -0.0048 | 0.0707 | 3.691E-09 | 0.0008 |
| rs10159261 | 1 | 15912987 | T | -0.0034 | 0.3599 | 4.842E-25 | 0.0003 |
| rs12061708 | 1 | 18809916 | A | -0.0026 | 0.2936 | 9.628E-14 | 0.0003 |
| rs2749153 | 1 | 23699340 | A | -0.0033 | 0.6894 | 7.838E-23 | 0.0003 |
| rs659437 | 1 | 46037394 | T | -0.0027 | 0.2184 | 3.315E-12 | 0.0004 |
| rs688540 | 1 | 48002447 | A | -0.003 | 0.8673 | 2.952E-08 | 0.0005 |
| rs17413465 | 1 | 55718708 | A | 0.0025 | 0.1824 | 8.933E-09 | 0.0004 |
| rs1757915 | 1 | 56615809 | A | 0.0021 | 0.3254 | 2.945E-10 | 0.0003 |
| rs679843 | 1 | 78707493 | T | 0.0021 | 0.334 | 5.214E-10 | 0.0003 |
| rs1887252 | 1 | 82957871 | C | -0.0019 | 0.6173 | 2.922E-09 | 0.0003 |
| rs7543734 | 1 | 94050911 | C | 0.0031 | 0.2056 | 9.898E-11 | 0.0005 |
| rs11166440 | 1 | 100808363 | A | 0.002 | 0.6026 | 1.838E-10 | 0.0003 |
| rs10857788 | 1 | 110012289 | A | 0.003 | 0.7001 | 1.961E-16 | 0.0004 |
| rs12736457 | 1 | 113258293 | C | 0.0054 | 0.8711 | 1.04E-25 | 0.0005 |
| rs3118119 | 1 | 150159616 | T | 0.003 | 0.1799 | 6.673E-11 | 0.0005 |
| rs267738 | 1 | 150940625 | T | -0.0048 | 0.7985 | 1.203E-32 | 0.0004 |
| rs4971100 | 1 | 155155731 | A | 0.002 | 0.4821 | 8.25E-10 | 0.0003 |
| rs3845534 | 1 | 163738950 | A | -0.0019 | 0.5336 | 1.198E-09 | 0.0003 |
| rs4656220 | 1 | 170649277 | T | 0.002 | 0.4224 | 3.292E-10 | 0.0003 |
| rs1011731 | 1 | 172346548 | A | -0.0019 | 0.5961 | 5.287E-09 | 0.0003 |
| rs3795503 | 1 | 180905694 | T | 0.002 | 0.3598 | 9.769E-10 | 0.0003 |
| rs78444298 | 1 | 184672098 | A | -0.0105 | 0.0185 | 4.886E-14 | 0.0014 |
| rs78329830 | 1 | 186769572 | A | -0.0054 | 0.9601 | 6.688E-09 | 0.0009 |
| rs3850625 | 1 | 201016296 | A | 0.0046 | 0.1159 | 1.135E-18 | 0.0005 |
| rs2808454 | 1 | 207231751 | A | 0.0019 | 0.5005 | 2.635E-09 | 0.0003 |
| rs75625374 | 1 | 208039431 | C | 0.0045 | 0.0582 | 4.479E-10 | 0.0007 |
| rs7535253 | 1 | 214744893 | T | 0.0021 | 0.2683 | 1.289E-09 | 0.0004 |
| rs2577134 | 1 | 220224321 | T | 0.002 | 0.6773 | 1.519E-08 | 0.0003 |
| rs61830291 | 1 | 221001142 | A | -0.0036 | 0.8991 | 1.202E-09 | 0.0006 |
| rs417237 | 1 | 228532195 | T | 0.0018 | 0.5671 | 7.504E-09 | 0.0003 |
| rs2490391 | 1 | 243469669 | A | -0.0024 | 0.4324 | 1.328E-14 | 0.0003 |
| rs3791221 | 2 | 226933 | A | 0.0022 | 0.6714 | 1.166E-11 | 0.0003 |
| rs807624 | 2 | 15782471 | T | 0.0032 | 0.4216 | 7.073E-23 | 0.0003 |
| rs4491726 | 2 | 18676276 | A | 0.0032 | 0.6934 | 5.484E-19 | 0.0004 |
| rs780093 | 2 | 27742603 | T | 0.0044 | 0.4171 | 1.627E-46 | 0.0003 |
| rs2301343 | 2 | 40680149 | T | -0.0023 | 0.7621 | 4.115E-10 | 0.0004 |
| rs10865189 | 2 | 43433257 | C | 0.0024 | 0.5126 | 3.291E-14 | 0.0003 |
| rs2971880 | 2 | 54885640 | A | -0.0024 | 0.3728 | 7.627E-15 | 0.0003 |
| rs10197255 | 2 | 67874553 | A | 0.0018 | 0.4045 | 1.151E-08 | 0.0003 |
| rs6546869 | 2 | 73895765 | A | 0.0059 | 0.2289 | 5.06E-48 | 0.0004 |
| rs11123169 | 2 | 113967075 | T | 0.0025 | 0.6769 | 9.24E-15 | 0.0003 |
| rs17050272 | 2 | 121306440 | A | -0.0022 | 0.4351 | 1.106E-12 | 0.0003 |
| rs11694902 | 2 | 121988884 | A | 0.0041 | 0.1379 | 1.063E-16 | 0.0005 |
| rs7425436 | 2 | 148759656 | A | 0.0024 | 0.6458 | 5.162E-13 | 0.0003 |
| rs4664475 | 2 | 152387553 | T | -0.002 | 0.378 | 4.633E-11 | 0.0003 |
| rs35472707 | 2 | 169995581 | T | -0.0073 | 0.0501 | 6.164E-19 | 0.0008 |
| rs187355703 | 2 | 176993583 | C | 0.01 | 0.9741 | 1.01E-18 | 0.0011 |
| rs35284526 | 2 | 178121524 | A | 0.0029 | 0.3237 | 6.241E-17 | 0.0003 |
| rs4666821 | 2 | 183077254 | T | 0.002 | 0.5295 | 2.5E-11 | 0.0003 |
| rs60980181 | 2 | 188168567 | A | -0.0027 | 0.165 | 1.754E-10 | 0.0004 |
| rs1047891 | 2 | 211540507 | A | -0.0065 | 0.2927 | 1.181E-75 | 0.0004 |
| rs1548945 | 2 | 217665788 | T | 0.0036 | 0.4389 | 8.395E-31 | 0.0003 |
| rs1050816 | 2 | 220358198 | T | 0.0026 | 0.333 | 1.116E-15 | 0.0003 |
| rs35669853 | 2 | 227287718 | A | 0.0024 | 0.1823 | 9.189E-09 | 0.0004 |
| rs13003198 | 2 | 234257105 | T | 0.0018 | 0.3693 | 3.137E-08 | 0.0003 |
| rs795009 | 3 | 12208671 | T | 0.002 | 0.7306 | 7.005E-09 | 0.0003 |
| rs6778731 | 3 | 13947504 | T | -0.0017 | 0.589 | 3.321E-08 | 0.0003 |
| rs6779998 | 3 | 30749965 | A | -0.0017 | 0.5174 | 1.648E-08 | 0.0003 |
| rs11914389 | 3 | 38527215 | T | 0.003 | 0.4815 | 3.111E-22 | 0.0003 |
| rs7651407 | 3 | 48443816 | T | 0.0025 | 0.4445 | 2.414E-11 | 0.0004 |
| rs4625 | 3 | 49572140 | A | -0.0023 | 0.6992 | 5.474E-11 | 0.0004 |
| rs2581820 | 3 | 53020544 | A | 0.0021 | 0.2864 | 7.915E-10 | 0.0003 |
| rs3774726 | 3 | 63974477 | T | -0.0021 | 0.363 | 3.654E-11 | 0.0003 |
| rs2289746 | 3 | 105455955 | T | -0.0019 | 0.4096 | 2.543E-09 | 0.0003 |
| rs9868185 | 3 | 121657593 | A | 0.0026 | 0.5016 | 5.028E-17 | 0.0003 |
| rs10934754 | 3 | 125906237 | T | 0.002 | 0.6039 | 1.326E-10 | 0.0003 |
| rs35320690 | 3 | 135932494 | T | -0.0025 | 0.7258 | 3.032E-11 | 0.0004 |
| rs7624084 | 3 | 141093285 | T | 0.0017 | 0.5767 | 1.967E-08 | 0.0003 |
| rs1397764 | 3 | 141750810 | A | 0.0043 | 0.274 | 2.509E-37 | 0.0003 |
| rs76272256 | 3 | 168888112 | T | 0.0024 | 0.2426 | 4.883E-10 | 0.0004 |
| rs56065557 | 3 | 185354216 | C | -0.0029 | 0.3244 | 4.32E-18 | 0.0003 |
| rs11919484 | 3 | 186432839 | T | -0.0026 | 0.3165 | 5.763E-16 | 0.0003 |
| rs9823161 | 3 | 193811168 | A | 0.0022 | 0.577 | 3.254E-09 | 0.0004 |
| rs75501914 | 4 | 3449781 | A | 0.0039 | 0.0941 | 8.979E-11 | 0.0006 |
| rs3775932 | 4 | 10090930 | A | -0.0018 | 0.5077 | 1.96E-09 | 0.0003 |
| rs16874073 | 4 | 23743962 | T | -0.0045 | 0.9474 | 6.561E-11 | 0.0007 |
| rs4864890 | 4 | 52686513 | T | -0.0023 | 0.3064 | 2.497E-09 | 0.0004 |
| rs28817415 | 4 | 77401452 | T | -0.0073 | 0.4037 | 3.49E-120 | 0.0003 |
| rs12509595 | 4 | 81182554 | T | -0.0035 | 0.7001 | 6.424E-25 | 0.0003 |
| rs223471 | 4 | 103698786 | C | 0.0028 | 0.3408 | 5.859E-19 | 0.0003 |
| rs55929207 | 4 | 109703549 | C | 0.0019 | 0.4776 | 3.425E-10 | 0.0003 |
| rs71606723 | 4 | 115498457 | A | 0.0025 | 0.7716 | 3.379E-12 | 0.0004 |
| rs13159523 | 5 | 676962 | A | -0.0024 | 0.4942 | 3.162E-13 | 0.0003 |
| rs13157326 | 5 | 34504277 | A | -0.0027 | 0.4493 | 1.667E-15 | 0.0003 |
| rs1362800 | 5 | 39378115 | T | -0.0049 | 0.3832 | 5.769E-51 | 0.0003 |
| rs495237 | 5 | 39950266 | T | 0.0027 | 0.2487 | 1.954E-14 | 0.0003 |
| rs11746506 | 5 | 44812566 | T | 0.0017 | 0.4088 | 3.004E-08 | 0.0003 |
| rs12520984 | 5 | 52787358 | C | 0.0019 | 0.3224 | 5.26E-09 | 0.0003 |
| rs79760705 | 5 | 53298716 | T | 0.0056 | 0.1076 | 6.471E-25 | 0.0005 |
| rs72759880 | 5 | 67750213 | T | -0.0056 | 0.1079 | 1.111E-26 | 0.0005 |
| rs2010352 | 5 | 68656327 | A | -0.0018 | 0.4534 | 1.74E-09 | 0.0003 |
| rs3797537 | 5 | 78322650 | A | 0.0019 | 0.7284 | 2.928E-08 | 0.0003 |
| rs12777 | 5 | 131671662 | C | 0.005 | 0.9586 | 1.071E-08 | 0.0009 |
| rs12163971 | 5 | 132226669 | A | -0.0029 | 0.1573 | 1.743E-12 | 0.0004 |
| rs11743174 | 5 | 148524820 | T | 0.0019 | 0.6671 | 1.336E-08 | 0.0003 |
| rs3812036 | 5 | 176813404 | T | -0.0065 | 0.2581 | 2.382E-74 | 0.0004 |
| rs11755724 | 6 | 7118990 | A | 0.0027 | 0.3721 | 1.133E-13 | 0.0004 |
| rs3765502 | 6 | 24354045 | T | 0.0024 | 0.7916 | 3.981E-08 | 0.0004 |
| rs144100226 | 6 | 34180297 | T | 0.0059 | 0.0367 | 5.982E-09 | 0.001 |
| rs13200335 | 6 | 41690823 | A | 0.0024 | 0.4626 | 8.058E-15 | 0.0003 |
| rs77915916 | 6 | 43287722 | A | 0.0046 | 0.917 | 7.253E-14 | 0.0006 |
| rs881858 | 6 | 43806609 | A | -0.0054 | 0.7138 | 2.924E-55 | 0.0003 |
| rs720989 | 6 | 44765535 | T | 0.0021 | 0.7879 | 1.839E-08 | 0.0004 |
| rs12212034 | 6 | 51492862 | T | -0.0018 | 0.3743 | 1.032E-08 | 0.0003 |
| rs6458868 | 6 | 52630153 | T | -0.002 | 0.6741 | 1.179E-09 | 0.0003 |
| rs3925003 | 6 | 55422618 | T | -0.0018 | 0.5804 | 1.966E-09 | 0.0003 |
| rs72912510 | 6 | 90118764 | A | -0.0024 | 0.1958 | 6.437E-09 | 0.0004 |
| rs1857859 | 6 | 100894587 | A | 0.0019 | 0.3107 | 2.583E-08 | 0.0003 |
| rs1268168 | 6 | 109008158 | A | 0.0024 | 0.3375 | 5.357E-14 | 0.0003 |
| rs7740107 | 6 | 130374461 | A | 0.0027 | 0.7445 | 8.929E-13 | 0.0004 |
| rs9375818 | 6 | 131882078 | A | -0.0031 | 0.2545 | 6.086E-18 | 0.0004 |
| rs3822939 | 6 | 133849789 | A | -0.0025 | 0.4222 | 2.164E-16 | 0.0003 |
| rs9397738 | 6 | 154986664 | A | 0.0027 | 0.838 | 3.497E-10 | 0.0004 |
| rs12207180 | 6 | 160633107 | A | -0.0085 | 0.113 | 2.635E-63 | 0.0005 |
| rs6968554 | 7 | 17287106 | A | -0.0019 | 0.4256 | 1.073E-09 | 0.0003 |
| rs3750081 | 7 | 32930876 | T | -0.0022 | 0.5931 | 1.874E-12 | 0.0003 |
| rs700753 | 7 | 46753684 | C | 0.0031 | 0.3166 | 2.117E-20 | 0.0003 |
| rs801193 | 7 | 66030612 | T | -0.002 | 0.5758 | 1.878E-09 | 0.0003 |
| rs41301394 | 7 | 75612803 | T | 0.0023 | 0.3176 | 3.654E-12 | 0.0003 |
| rs6973656 | 7 | 77422583 | A | 0.0035 | 0.6359 | 5.663E-28 | 0.0003 |
| rs35154268 | 7 | 127505755 | A | -0.0022 | 0.7187 | 9.854E-09 | 0.0004 |
| rs3757387 | 7 | 128576086 | T | 0.003 | 0.5895 | 6.961E-20 | 0.0003 |
| rs62491533 | 7 | 129564134 | T | -0.0027 | 0.8097 | 1.079E-11 | 0.0004 |
| rs10254101 | 7 | 151415536 | T | -0.0068 | 0.2768 | 1.847E-67 | 0.0004 |
| rs868822 | 7 | 156252939 | T | 0.0029 | 0.3331 | 2.201E-18 | 0.0003 |
| rs1533059 | 8 | 8684953 | A | 0.0025 | 0.5142 | 1.189E-14 | 0.0003 |
| rs7832708 | 8 | 10190040 | T | 0.0022 | 0.4947 | 9.786E-10 | 0.0004 |
| rs10098664 | 8 | 11417493 | T | -0.0021 | 0.4859 | 6.125E-10 | 0.0003 |
| rs34861762 | 8 | 23748420 | T | -0.0043 | 0.3863 | 4.06E-41 | 0.0003 |
| rs10102889 | 8 | 32435620 | C | -0.0036 | 0.509 | 6.654E-09 | 0.0006 |
| rs2976178 | 8 | 87332552 | C | -0.0025 | 0.6717 | 7.807E-14 | 0.0003 |
| rs2954017 | 8 | 126476873 | T | 0.0024 | 0.4575 | 1.656E-12 | 0.0003 |
| rs12377027 | 9 | 20554583 | A | -0.0026 | 0.8182 | 2.886E-08 | 0.0005 |
| rs13287724 | 9 | 33169034 | A | -0.003 | 0.8913 | 4.665E-08 | 0.0006 |
| rs544169 | 9 | 33956791 | A | 0.0022 | 0.7305 | 3.885E-11 | 0.0003 |
| rs2039424 | 9 | 71432174 | A | 0.0044 | 0.6356 | 2.064E-44 | 0.0003 |
| rs1321917 | 9 | 119324929 | C | -0.0023 | 0.4364 | 1.382E-13 | 0.0003 |
| rs7024579 | 9 | 139100413 | T | 0.0023 | 0.2889 | 8.166E-11 | 0.0004 |
| rs80282103 | 10 | 899071 | A | 0.0078 | 0.9143 | 1.229E-44 | 0.0006 |
| rs6481598 | 10 | 29781798 | C | 0.0024 | 0.7934 | 1.51E-09 | 0.0004 |
| rs7072591 | 10 | 35150364 | A | 0.0019 | 0.555 | 2.669E-09 | 0.0003 |
| rs8474 | 10 | 51026705 | C | 0.002 | 0.4495 | 1.77E-09 | 0.0003 |
| rs10821905 | 10 | 52646093 | A | 0.0037 | 0.1845 | 9.386E-19 | 0.0004 |
| rs10821944 | 10 | 63785089 | T | 0.002 | 0.7045 | 3.885E-09 | 0.0003 |
| rs7475348 | 10 | 69965177 | T | 0.0031 | 0.4595 | 1.236E-22 | 0.0003 |
| rs12240572 | 10 | 75016365 | A | -0.0032 | 0.1671 | 5.252E-09 | 0.0006 |
| rs816850 | 10 | 79252446 | C | -0.002 | 0.2532 | 7.402E-09 | 0.0004 |
| rs7095954 | 10 | 82209232 | A | -0.0018 | 0.4536 | 3.737E-08 | 0.0003 |
| rs9420446 | 10 | 88880689 | T | 0.0023 | 0.2679 | 4.973E-08 | 0.0004 |
| rs2068888 | 10 | 94839642 | A | -0.0024 | 0.4907 | 4.426E-15 | 0.0003 |
| rs4918943 | 10 | 97278922 | A | -0.0022 | 0.2185 | 9.186E-09 | 0.0004 |
| rs284859 | 10 | 104573017 | T | 0.0026 | 0.2072 | 4.986E-12 | 0.0004 |
| rs1536225 | 10 | 105202318 | T | -0.0021 | 0.6328 | 1.913E-11 | 0.0003 |
| rs1055256 | 10 | 126446592 | A | 0.0025 | 0.4235 | 3.561E-16 | 0.0003 |
| rs11564722 | 11 | 2178330 | T | 0.0033 | 0.311 | 2.119E-20 | 0.0004 |
| rs63934 | 11 | 2789062 | A | 0.0041 | 0.8265 | 3.867E-23 | 0.0004 |
| rs1541937 | 11 | 5578558 | A | -0.0029 | 0.6395 | 5.179E-17 | 0.0004 |
| rs963837 | 11 | 30749090 | T | -0.0057 | 0.5728 | 4.324E-73 | 0.0003 |
| rs6484504 | 11 | 31424823 | T | -0.0026 | 0.3053 | 6.449E-15 | 0.0003 |
| rs61897431 | 11 | 47427667 | T | 0.0029 | 0.6472 | 6.331E-16 | 0.0004 |
| rs2727040 | 11 | 49057603 | T | -0.0026 | 0.2382 | 2.156E-09 | 0.0004 |
| rs1813937 | 11 | 50468801 | T | 0.0022 | 0.7185 | 6.886E-10 | 0.0004 |
| rs1783827 | 11 | 57409538 | A | -0.002 | 0.6021 | 3.769E-09 | 0.0003 |
| rs948493 | 11 | 65552154 | T | -0.0033 | 0.3306 | 1.979E-24 | 0.0003 |
| rs3892895 | 11 | 68884755 | A | -0.0023 | 0.3993 | 6.172E-13 | 0.0003 |
| rs11237450 | 11 | 78023356 | A | 0.0032 | 0.254 | 1.486E-14 | 0.0004 |
| rs6589750 | 11 | 119326726 | A | 0.002 | 0.6315 | 1.539E-09 | 0.0003 |
| rs10790452 | 11 | 121584931 | T | 0.002 | 0.7369 | 7.138E-09 | 0.0003 |
| rs11062167 | 12 | 364739 | A | -0.0039 | 0.4834 | 2.483E-34 | 0.0003 |
| rs632887 | 12 | 3392351 | A | 0.0032 | 0.6157 | 2.193E-24 | 0.0003 |
| rs4238020 | 12 | 4616642 | T | 0.0029 | 0.8703 | 8.407E-09 | 0.0005 |
| rs117113238 | 12 | 12209203 | A | 0.0039 | 0.0939 | 8.604E-11 | 0.0006 |
| rs10846157 | 12 | 15325031 | A | -0.0034 | 0.7602 | 8.34E-21 | 0.0004 |
| rs2634675 | 12 | 48740855 | A | 0.0025 | 0.52 | 2.66E-13 | 0.0003 |
| rs12313306 | 12 | 57751854 | T | 0.0029 | 0.229 | 1.456E-14 | 0.0004 |
| rs1275609 | 12 | 76271183 | A | 0.0024 | 0.3862 | 4.606E-13 | 0.0003 |
| rs41284816 | 13 | 50655989 | T | -0.0078 | 0.0262 | 1.717E-10 | 0.0012 |
| rs500830 | 13 | 72348768 | T | 0.0029 | 0.4603 | 1.962E-19 | 0.0003 |
| rs72683923 | 14 | 50735947 | T | -0.0074 | 0.9791 | 3.368E-08 | 0.0013 |
| rs6574652 | 14 | 81870100 | T | -0.0017 | 0.5016 | 2.413E-08 | 0.0003 |
| rs1028455 | 14 | 88829975 | A | 0.002 | 0.3273 | 4.78E-10 | 0.0003 |
| rs17184313 | 14 | 93102251 | T | -0.0029 | 0.1744 | 1.978E-10 | 0.0005 |
| rs61993680 | 14 | 100752644 | A | -0.0019 | 0.6082 | 1.48E-08 | 0.0003 |
| rs12913015 | 15 | 39305443 | T | 0.0027 | 0.4148 | 2.284E-17 | 0.0003 |
| rs6492982 | 15 | 41399951 | T | -0.0033 | 0.5569 | 3.109E-20 | 0.0004 |
| rs1145077 | 15 | 45683795 | T | -0.0085 | 0.4206 | 6.85E-142 | 0.0003 |
| rs690428 | 15 | 53950578 | A | -0.0039 | 0.6653 | 8.234E-32 | 0.0003 |
| rs1994887 | 15 | 57793765 | A | -0.002 | 0.257 | 1.605E-08 | 0.0004 |
| rs956006 | 15 | 62808539 | T | 0.0019 | 0.3309 | 4.448E-09 | 0.0003 |
| rs11071738 | 15 | 63580155 | T | -0.0025 | 0.5726 | 2.013E-15 | 0.0003 |
| rs11071939 | 15 | 67463391 | T | -0.0039 | 0.9286 | 4.918E-10 | 0.0006 |
| rs351237 | 15 | 74477239 | A | -0.0018 | 0.62 | 4.418E-08 | 0.0003 |
| rs2472297 | 15 | 75027880 | T | 0.0039 | 0.2567 | 4.467E-20 | 0.0004 |
| rs4886696 | 15 | 75664570 | A | -0.0032 | 0.333 | 2.031E-19 | 0.0004 |
| rs4886755 | 15 | 76298132 | A | 0.0041 | 0.4971 | 2.042E-39 | 0.0003 |
| rs166906 | 15 | 76802175 | T | 0.0033 | 0.0902 | 8.688E-10 | 0.0005 |
| rs17507300 | 15 | 83722059 | A | 0.0024 | 0.8319 | 1.048E-08 | 0.0004 |
| rs7169629 | 15 | 85191274 | C | 0.0018 | 0.4771 | 1.623E-08 | 0.0003 |
| rs59646751 | 15 | 99276521 | T | -0.0023 | 0.3043 | 3.061E-12 | 0.0003 |
| rs438339 | 16 | 2003425 | T | 0.0035 | 0.8818 | 4.967E-08 | 0.0006 |
| rs1635404 | 16 | 3747042 | T | -0.0025 | 0.7073 | 5.681E-11 | 0.0004 |
| rs193538 | 16 | 16127916 | T | -0.002 | 0.6993 | 1.818E-09 | 0.0003 |
| rs77924615 | 16 | 20392332 | A | 0.0098 | 0.2033 | 1.45E-138 | 0.0004 |
| rs9932625 | 16 | 51735746 | A | -0.003 | 0.2566 | 2.242E-17 | 0.0003 |
| rs7203398 | 16 | 53189672 | A | 0.0025 | 0.7412 | 4.668E-13 | 0.0003 |
| rs7185391 | 16 | 68323115 | T | -0.0027 | 0.2735 | 8.559E-14 | 0.0004 |
| rs62050038 | 16 | 69802865 | A | 0.0028 | 0.8257 | 1.261E-11 | 0.0004 |
| rs62053077 | 16 | 71643669 | T | -0.0021 | 0.4289 | 3.722E-09 | 0.0004 |
| rs1858800 | 16 | 73024276 | T | 0.002 | 0.3219 | 2.095E-09 | 0.0003 |
| rs28581385 | 16 | 79942679 | A | -0.0028 | 0.8358 | 1.352E-11 | 0.0004 |
| rs154656 | 16 | 89708003 | A | -0.003 | 0.4269 | 4.107E-18 | 0.0003 |
| rs28735420 | 17 | 12139964 | T | 0.0039 | 0.9209 | 8.754E-10 | 0.0006 |
| rs2349648 | 17 | 17017267 | T | -0.0017 | 0.3387 | 4.925E-08 | 0.0003 |
| rs9891340 | 17 | 17543846 | T | 0.0024 | 0.5517 | 2.744E-11 | 0.0004 |
| rs2440165 | 17 | 19428719 | T | 0.004 | 0.6364 | 1.722E-31 | 0.0003 |
| rs2411192 | 17 | 34882998 | A | -0.0024 | 0.596 | 2.14E-15 | 0.0003 |
| rs4794813 | 17 | 37670994 | A | 0.0055 | 0.2439 | 3.577E-53 | 0.0004 |
| rs227731 | 17 | 54773238 | T | 0.0018 | 0.5672 | 1.437E-08 | 0.0003 |
| rs35662455 | 17 | 56755223 | C | 0.003 | 0.8855 | 3.858E-08 | 0.0005 |
| rs9903801 | 17 | 58915261 | C | 0.0047 | 0.1554 | 1.014E-27 | 0.0004 |
| rs9895661 | 17 | 59456589 | T | 0.0069 | 0.7223 | 2.49E-71 | 0.0004 |
| rs8866 | 17 | 65373979 | C | -0.0018 | 0.622 | 2.167E-08 | 0.0003 |
| rs883541 | 17 | 66449122 | A | -0.0022 | 0.7176 | 2.657E-10 | 0.0003 |
| rs1719934 | 18 | 5585158 | A | 0.0026 | 0.5752 | 2.617E-17 | 0.0003 |
| rs16942751 | 18 | 24393213 | A | -0.0029 | 0.1821 | 2.209E-09 | 0.0005 |
| rs4940525 | 18 | 59354616 | T | 0.0025 | 0.2603 | 4.068E-13 | 0.0003 |
| rs8096658 | 18 | 77156537 | C | 0.005 | 0.5543 | 3.149E-44 | 0.0004 |
| rs2974751 | 19 | 13053034 | A | 0.0018 | 0.376 | 4.385E-08 | 0.0003 |
| rs8101667 | 19 | 33402419 | T | 0.0044 | 0.3933 | 9.349E-44 | 0.0003 |
| rs7251730 | 19 | 36997147 | T | 0.0024 | 0.3452 | 2.197E-12 | 0.0003 |
| rs113445505 | 19 | 38157969 | T | 0.0037 | 0.3564 | 6.963E-27 | 0.0003 |
| rs281380 | 19 | 49214470 | T | -0.0021 | 0.5628 | 2.86E-10 | 0.0003 |
| rs34647824 | 19 | 50138143 | A | -0.0021 | 0.7386 | 3.953E-08 | 0.0004 |
| rs62187537 | 20 | 1333060 | T | 0.0039 | 0.0684 | 9.206E-09 | 0.0007 |
| rs1509117 | 20 | 8303120 | A | 0.0024 | 0.3018 | 7.066E-10 | 0.0004 |
| rs1041606 | 20 | 14677788 | T | -0.0021 | 0.2319 | 2.516E-08 | 0.0004 |
| rs2273684 | 20 | 33529766 | T | 0.0032 | 0.5756 | 7.791E-25 | 0.0003 |
| rs17216707 | 20 | 52732362 | T | -0.0051 | 0.8134 | 1.068E-33 | 0.0004 |
| rs2235826 | 20 | 56143169 | A | -0.003 | 0.7932 | 6.825E-15 | 0.0004 |
| rs1407040 | 20 | 57472174 | T | 0.0018 | 0.6756 | 1.442E-08 | 0.0003 |
| rs35636653 | 20 | 60858758 | T | 0.0022 | 0.3369 | 2.276E-11 | 0.0003 |
| rs72629024 | 20 | 62152519 | C | 0.0035 | 0.6634 | 2.019E-13 | 0.0005 |
| rs4408777 | 20 | 62706105 | A | -0.0021 | 0.5139 | 5.404E-11 | 0.0003 |
| rs2823139 | 21 | 16576783 | A | -0.0026 | 0.3274 | 5.235E-16 | 0.0003 |
| rs2834317 | 21 | 35356706 | A | -0.0035 | 0.1425 | 4.256E-14 | 0.0005 |
| rs2244237 | 21 | 37818141 | T | 0.0027 | 0.2191 | 6.275E-11 | 0.0004 |
| rs131263 | 22 | 30133045 | T | 0.0024 | 0.4584 | 2.201E-11 | 0.0004 |
| rs80576 | 22 | 36539804 | A | -0.0028 | 0.1592 | 1.268E-09 | 0.0005 |
| rs4820324 | 22 | 38599857 | C | -0.0023 | 0.5902 | 5.067E-14 | 0.0003 |
| rs112880707 | 22 | 40884662 | T | 0.0052 | 0.1713 | 4.876E-31 | 0.0005 |
| rs738527 | 22 | 43112961 | T | 0.0032 | 0.2917 | 4.175E-21 | 0.0003 |

Abbreviation: SNP, single nucleotide polymorphism; CHR, chromosome; BP,base position; EAF, effect allele frequency.

**Supplemental Table 3. The percentages of participants with missing covariates.**

| **Characteristic** | **Percentage of missing data** |
| --- | --- |
| Sex | 0.00% |
| Age | 0.00% |
| Year of birth | 0.00% |
| Race | 0.38% |
| Cigarette smoking | 0.07% |
| Alcohol consumption | 0.08% |
| Education level | 0.48% |
| Townsend deprivation index | 0.11% |
| BMI | 0.27% |
| Creatinine | 5.60% |
| Total sugars intake per day | 0.00% |
| Fat intake per day | 0.00% |
| Protein intake per day | 0.00% |
| Carbohydrate intake per day | 0.00% |
| Coffee intake per day | 0.00% |
| Tea intake per day | 0.00% |
| Cereal intake per day | 0.00% |
| Total Metabolic Equivalent Task minutes per week | 16.27% |
| Diabetes history | 0.00% |
| Hypertension history | 0.00% |
| Dyslipidemia history | 0.00% |

Abbreviations: BMI, body mass index.

**Supplemental Table 4. Characteristics of participants in this study and all participants in UK Biobank.**

| **Characteristics** | **All participants in UK Biobank** | **Participants in this study** | **P value** ^a^ |
| --- | --- | --- | --- |
|  | **N=502,369** | **N=156,000** |  |
| **Age (year, mean (SD))** | 57.0 (8.1) | 56.8 (7.9) | <0.001 |
| **Gender, Female, n (%)** | 273301 (54.4) | 85020 (54.5) | 0.50 |
| **Race, n(%)** |  |  | <0.001 |
| **White** | 472573 (94.6) | 148843 (95.8) |  |
| **Non-white** | 27018 (5.4) | 6565 (4.2) |  |
| **Cigarette smoking, n (%)** |  |  | <0.001 |
| **Never** | 273401 (54.7) | 88975 (57.2) |  |
| **Previous** | 173009 (34.6) | 54316 (34.9) |  |
| **Current** | 52961 (10.6) | 12293 (7.9) |  |
| **Alcohol consumption, n (%)** |  |  | <0.001 |
| **Not current** | 40624 (8.1) | 9267 (5.9) |  |
| **Two or fewer times a week** | 243084 (48.5) | 69575 (44.6) |  |
| **Three or more times a week** | 217159 (43.4) | 77032 (49.4) |  |
| **Education level, n (%)** |  |  | <0.001 |
| **No qualification** | 85257 (17.3) | 12846 (8.3) |  |
| **Any other qualification** | 245876 (50.0) | 73730 (47.5) |  |
| **Degree or above** | 161104 (32.7) | 68677 (44.2) |  |
| **Townsend deprivation index, (mean (SD))** | -1.3 (3.1) | -1.6 (2.9) | <0.001 |
| **BMI (kg/m^2^, mean (SD))** | 27.4 (4.8) | 26.4 (4.3) | <0.001 |
| **BMI category (kg/m^2^), n (%)** |  |  | <0.001 |
| **< 25** | 164978 (33.0) | 64010 (41.1) |  |
| **25 - 30** | 212062 (42.5) | 64340 (41.4) |  |
| **≥ 30** | 122222 (24.5) | 27222 (17.5) |  |
| **eGFR, (ml/min/1.73m^2^, mean (SD))** | 94.3 (13.1) | 95.5 (11.4) | <0.001 |
| **Diabetes history, n (%)** | 47244 (9.4) | 9126 (5.9) | <0.001 |
| **Hypertension history, n (%)** | 251955 (50.2) | 71982 (46.1) | <0.001 |
| **Dyslipidemia history, n (%)** | 227167 (45.2) | 68223 (43.7) | <0.001 |

Abbreviation: eGFR, estimated glomerular filtration rate; BMI, body-mass index; SD, standard deviation.

Townsend deprivation index composite area-level measure of deprivation based on unemployment, non-car ownership, non-home ownership, and household overcrowding; a higher score indicates higher deprivation.

^a^ Group comparisons were executed using analysis of variance (ANOVA) for normally distributed variables, the Mann-Whitney U test for non-parametric variables, and either Fisher’s exact test or the chi-squared test for categorical variables.

**Supplemental Table 5. Association between the per SD increase of artificial sweetener intake and incidence of CKD in the longitudinal cohort.**

| **Exposure** | **Crude** | **Model l^a^** | **Model 2^b^** | **Model 3^c^** |
| --- | --- | --- | --- | --- |
|  | **HR (95% CI); *P value*** | **HR (95% CI); *P value*** | **HR (95% CI); *P value*** | **HR (95% CI); *P value*** |
| **per SD increase of AS intake** | 1.13  (1.11,1.15) | 1.09  (1.07,1.11) | 1.05  (1.03,1.07) | 1.04  (1.02,1.06) |
|  | <0.001 | <0.001 | <0.001 | <0.001 |

Abbreviation: CKD, chronic kidney disease; AS, artificial sweetener; eGFR, estimated glomerular filtration rate; BMI, body-mass index; SD, standard deviation; HR, hazard ratio; CI, confidence interval.

^a^ Model 1 the adjustment factors included age, sex and race.

^b^ Model 2 the adjustment factors included age, sex , race, BMI, eGFR smoking, drinking, education level, townsend deprivation index and total Metabolic Equivalent Task (MET) minutes per week.

^c^ Model 3 the adjustment factors included age, sex , race, BMI, eGFR smoking, drinking, education level, townsend deprivation index, total Metabolic Equivalent Task (MET) minutes per week,coffee intake, tea intake, cereal intake, protein intake, carbohydrate intake, energy intake, total sugars intake, fat intake and comorbidities (history of hypertension, history of diabetes and history of dyslipidemia).

**Supplemental Table 6. Association between artificial sweetener intake and eGFR slope in participants with follow-up serum creatinine measurement.**

| **AS intake** | **Sample size** | **eGFR slope** | **Crude** | Model l^a^ | Model 2^b^ | Model 3^c^ |
| --- | --- | --- | --- | --- | --- | --- |
|  |  | **Median(IQR)** | Beta (95% CI); P | Beta (95% CI); P | Beta (95% CI); P | Beta (95% CI); P |
| **None-AS** | 41590 | -3.18 (-6.74,-1.43) | Ref | Ref | Ref | Ref |
| **Low-AS** | 2301 | -3.49 (-7.51,-1.59) | -0.45 (-0.78,-0.13) | -0.34 (-0.67,-0.02) | -0.16 (-0.49,0.16) | -0.09 (-0.42,0.23) |
|  |  |  | 0.006 | 0.04 | 0.33 | 0.58 |
| **High-AS** | 2636 | -3.60 (-7.92,-1.68) | -0.67 (-0.98,-0.37) | -0.61 (-0.92,-0.31) | -0.38 (-0.69,-0.08) | -0.34 (-0.65,-0.03) |
|  |  |  | <0.001 | <0.001 | 0.01 | 0.03 |

Abbreviation: eGFR, estimated glomerular filtration rate; BMI, body-mass index; AS, artificial sweetener; IQR, interquartile range; CI, confidence interval; Ref, reference.

^a^ Model 1 the adjustment factors included age, sex and race.

^b^ Model 2 the adjustment factors included age, sex, race, BMI, eGFR, smoking, drinking, education level, townsend deprivation index and total Metabolic Equivalent Task (MET) minutes per week.

^c^ Model 3 the adjustment factors included age, sex, race, BMI, eGFR, smoking, drinking, education level, townsend deprivation index, total Metabolic Equivalent Task (MET) minutes per week, coffee intake, tea intake, cereal intake, protein intake, carbohydrate intake, energy intake, total sugars intake, fat intake and comorbidities (history of hypertension, history of diabetes and history of dyslipidemia).

**Supplemental Table 7. Association between artificial sweeteners intake and time to incident CKD in the subcohort that included participants developed CKD with follow-up.**

| **AS intake** | **Sample size** | **Time to CKD (years)** | **Crude** | Model l^a^ | Model 2^b^ | Model 3^c^ |
| --- | --- | --- | --- | --- | --- | --- |
|  |  | **Median (IQR)** | Beta (95% CI); P | Beta (95% CI); P | Beta (95% CI); P | Beta (95% CI); P |
| **None-AS** | 5113 | 7.01 (3.87,10.72) | Ref | Ref | Ref | Ref |
| **Low-AS** | 383 | 7.11 (4.17,10.79) | 0.14 (-0.28,0.55) | 0.18 (-0.23,0.60) | 0.14 (-0.28,0.55) | 0.10 (-0.31,0.52) |
|  |  |  | 0.52 | 0.39 | 0.52 | 0.63 |
| **High-AS** | 526 | 7.67 (4.16,10.84) | 0.20 (-0.16,0.56) | 0.15 (-0.21,0.51) | 0.07 (-0.29,0.43) | 0.05 (-0.31,0.42) |
|  |  |  | 0.29 | 0.42 | 0.70 | 0.78 |

Abbreviation: CKD, chronic kidney disease; AS, artificial sweetener; eGFR, estimated glomerular filtration rate; BMI, body-mass index; IQR, interquartile range; CI, confidence interval; Ref, reference.

^a^ Model 1 the adjustment factors included age, sex and race.

^b^ Model 2 the adjustment factors included age, sex, race, BMI, eGFR, smoking, drinking, education level, townsend deprivation index and total Metabolic Equivalent Task (MET) minutes per week.

^c^ Model 3 the adjustment factors included age, sex, race, BMI, eGFR, smoking, drinking, education level, townsend deprivation index, total Metabolic Equivalent Task (MET) minutes per week,coffee intake, tea intake, cereal intake, protein intake, carbohydrate intake, energy intake, total sugars intake, fat intake and comorbidities (history of hypertension, history of diabetes and history of dyslipidemia).

**Supplemental Table 8. Substitution analysis of the association between risk of new-onset CKD and artificial sweeteners to replace sugar.**

| **Substitution analysis** | **Crude** | Model l^a^ | Model 2^b^ | Model 3^c^ |
| --- | --- | --- | --- | --- |
|  | HR (95% CI); P value | HR (95% CI); P value | HR (95% CI); P value | HR (95% CI); P value |
| **Sugar intake** | Ref | Ref | Ref | Ref |
| **Artificial sweeteners intake** | 1.06  (1.05,1.07) | 1.05  (1.04,1.07) | 1.03  (1.02,1.04) | 1.02  (1.01,1.04) |
|  | <0.001 | <0.001 | <0.001 | <0.001 |

Abbreviation: CKD, chronic kidney disease; eGFR, estimated glomerular filtration rate; BMI, body-mass index; HR, hazard ratio; CI, confidence interval; Ref, reference.

^a^ Model 1 the adjustment factors included age, sex and race.

^b^ Model 2 the adjustment factors included age, sex, race, BMI, eGFR, smoking, drinking, education level, townsend deprivation index and total Metabolic Equivalent Task (MET) minutes per week.

^c^ Model 3 the adjustment factors included age, sex, race, BMI, eGFR, smoking, drinking, education level, townsend deprivation index, total Metabolic Equivalent Task (MET) minutes per week,coffee intake, tea intake, cereal intake, protein intake, carbohydrate intake, energy intake, fat intake and comorbidities (history of hypertension, history of diabetes and history of dyslipidemia).

**Supplemental Table 9. Associations of CKD genetic risk score with the risk of incident CKD in the longitudinal cohort.**

| **Genetic risk** | **Cases/Total** | **IR (95% CI)** | **Crude** | **Model 1** | **Model 2** | **Model 3** |
| --- | --- | --- | --- | --- | --- | --- |
|  |  | **1000 Person-years** | **HR (95% CI); *P*** | **HR (95% CI); *P*** | **HR (95% CI); *P*** | **HR (95% CI); *P*** |
| **Low-PRS** | 1410/47764 | 2.22  (2.11,2.34) | Ref | Ref | Ref | Ref |
| **Intermediate-PRS** | 1726/47759 | 2.72  (2.60,2.85) | 1.23 (1.14,1.32) | 1.23 (1.15,1.32) | 1.09 (1.02,1.17) | 1.10 (1.02,1.18) |
|  |  |  | <0.001 | <0.001 | 0.01 | 0.01 |
| **High-PRS** | 2387/47776 | 3.79  (3.64,3.94) | 1.71 (1.60,1.82) | 1.73 (1.62,1.85) | 1.34 (1.26,1.44) | 1.35 (1.27,1.45) |
|  |  |  | <0.001 | <0.001 | <0.001 | <0.001 |

Abbreviation: CKD, chronic kidney disease; PRS, polygenic risk score; IR, incidence rate; eGFR, estimated glomerular filtration rate; BMI, body-mass index; HR, hazard ratio; CI, confidence interval; Ref, reference.

^a^ Model 1 the adjustment factors included age, sex and race.

^b^ Model 2 the adjustment factors included age, sex, race, BMI, eGFR, smoking, drinking, education level, townsend deprivation index and total Metabolic Equivalent Task (MET) minutes per week.

^c^ Model 3 the adjustment factors included age, sex, race, BMI, eGFR, smoking, drinking, education level, townsend deprivation index, total Metabolic Equivalent Task (MET) minutes per week,coffee intake, tea intake, cereal intake, protein intake, carbohydrate intake, energy intake, total sugars intake, fat intake and comorbidities (history of hypertension, history of diabetes and history of dyslipidemia).

**Supplemental Table 10. Characteristics of all participants in the longitudinal cohort after PSM.**

| **Characteristics** | **Overall** | **None-AS** | **High-AS** | **SMD** ^a^ |
| --- | --- | --- | --- | --- |
|  | **N=40,800** | **N=32,640** | **N=8,160** |  |
| **Age (year, mean (SD))** | 58.5 (7.5) | 58.5 (7.5) | 58.4 (7.5) | 0.01 |
| **Gender, Female, n (%)** | 20613 (50.5) | 16511 (50.6) | 4102 (50.3) | 0.01 |
| **Race, n(%)** |  |  |  | 0.007 |
| **White** | 39219 (96.5) | 31369 (96.5) | 7850 (96.6) |  |
| **Non-white** | 1431 (3.5) | 1153 (3.5) | 278 (3.4) |  |
| **Cigarette smoking, n (%)** |  |  |  | 0.008 |
| **Never** | 17023 (41.8) | 13623 (41.8) | 3400 (41.8) |  |
| **Previous** | 18404 (45.2) | 14741 (45.3) | 3663 (45.0) |  |
| **Current** | 5273 (13.0) | 4201 (12.9) | 1072 (13.2) |  |
| **Alcohol consumption, n (%)** |  |  |  | 0.008 |
| **Not current** | 3357 (8.2) | 2675 (8.2) | 682 (8.4) |  |
| **Two or fewer times a week** | 21133 (51.8) | 16926 (51.9) | 4207 (51.6) |  |
| **Three or more times a week** | 16284 (39.9) | 13014 (39.9) | 3270 (40.1) |  |
| **Education level, n (%)** |  |  |  | 0.02 |
| **No qualification** | 6035 (14.9) | 4799 (14.8) | 1236 (15.2) |  |
| **Any other qualification** | 23574 (58.1) | 18931 (58.4) | 4643 (57.2) |  |
| **Degree or above** | 10946 (27.0) | 8710 (26.8) | 2236 (27.6) |  |
| **Townsend deprivation index, (mean (SD))** | -1.5 (2.9) | -1.5 (2.9) | -1.4 (2.9) | 0.02 |
| **BMI (kg/m^2^, mean (SD))** | 28.0 (4.5) | 27.9 (4.5) | 28.1 (4.6) | 0.04 |
| **BMI category (kg/m^2^), n (%)** |  |  |  | 0.03 |
| **< 25** | 9723 (23.9) | 7718 (23.7) | 2005 (24.7) |  |
| **25 - 30** | 19581 (48.2) | 15741 (48.4) | 3840 (47.2) |  |
| **≥ 30** | 11362 (27.9) | 9075 (27.9) | 2287 (28.1) |  |
| **eGFR (ml/min/1.73m^2^, mean (SD))** | 94.0 (11.5) | 94.1 (11.5) | 93.9 (11.5) | 0.01 |
| **Artificial sweetener intake (teaspoon/day, mean (SD))** | 1.5 (3.3) | 0.0 (0.0) | 7.3 (3.5) | 2.96 |
| **Total sugars intake (g/day, mean (SD))** | 125.1 (51.4) | 125.1 (51.2) | 125.1 (52.3) | <0.001 |
| **Diabetes history, n (%)** | 4697 (11.5) | 3722 (11.4) | 975 (11.9) | 0.02 |
| **Hypertension history, n (%)** | 22105 (54.2) | 17738 (54.3) | 4367 (53.5) | 0.02 |
| **Dyslipidemia history, n (%)** | 19015 (46.6) | 15238 (46.7) | 3777 (46.3) | 0.008 |

Abbreviation: eGFR, estimated glomerular filtration rate; BMI, body-mass index; SD, standard deviation. SMD, standardized mean difference

Townsend deprivation index composite area-level measure of deprivation based on unemployment, non-car ownership, non-home ownership, and household overcrowding; a higher score indicates higher deprivation.

^a^ SMD be used to compare the mean of baseline covariate between None-AS and High-AS group.

**Supplemental Table 11. Sensitivity analysis I of the association between artificial sweeteners intake and the incidence of CKD after PSM.**

| **Artificial sweetener intake** | **Cases/Total** | **IR (95% CI)** | **Sensitivity analysis I** | |
| --- | --- | --- | --- | --- |
|  |  | **1000 Person-years** | **HR (95% CI)** | P value |
| **None-AS** | 1754/32640 | 4.10  (3.91,4.29) | Ref | - |
| **High-AS** | 517/8160 | 4.85  (4.43,5.27) | 1.09  (1.04,1.14) | <0.001 |

Abbreviation: CKD, chronic kidney disease; AS, artificial sweetener; IR, incidence rate; HR, hazard ratio; CI, confidence interval; Ref, reference.

S**upplemental Table 12. Sensitivity analysis II-V of the association between artificial sweeteners intake and the incidence of CKD.**

| **AS intake** | **Cases/Total** | **IR (95% CI)**  **1000 Person-years** | **Sensitivity analysis**  **II ^a^** | **Sensitivity analysis**  **III ^b^** | **Sensitivity analysis**  **IV ^c^** | **Sensitivity analysis**  **V ^d^** |
| --- | --- | --- | --- | --- | --- | --- |
|  |  |  | **HR (95% CI);** P | **HR (95% CI);** P | **HR (95% CI);** P | **HR (95% CI);** P |
| **None-AS** | 5113/140196 | 2.75 (2.68,2.83) | Ref | Ref | Ref | Ref |
| **Low-AS** | 383/7565 | 3.85 (3.46,4.23) | 1.05 (0.94,1.16) | 1.05 (0.94,1.16) | 1.04 (0.94,1.15) | 1.05 (0.94,1.16) |
|  |  |  | 0.40 | 0.39 | 0.48 | 0.41 |
| **High-AS** | 526/8239 | 4.89 (4.47,5.30) | 1.18 (1.08,1.29) | 1.19 (1.08,1.30) | 1.18 (1.08,1.29) | 1.19 (1.08,1.30) |
|  |  |  | <0.001 | <0.001 | <0.001 | <0.001 |

Abbreviation: CKD, chronic kidney disease; AS, artificial sweetener; eGFR, estimated glomerular filtration rate; BMI, body-mass index; IR, incidence rate; HR, hazard ratio; CI, confidence interval; Ref, reference.

^a^ In Sensitivity analysis II, the adjustment factors included age, sex, race, BMI, eGFR, smoking, drinking, education level, townsend deprivation index, total Metabolic Equivalent Task (MET) minutes per week,coffee intake, tea intake, cereal intake, protein intake, carbohydrate intake, energy intake, total sugars intake, fat intake, comorbidities (history of hypertension, diabetes and dyslipidemia), red and processed meat intake, vegetable intake and fruit intake.

^b^ In Sensitivity analysis III, the adjustment factors included age, sex, race, BMI, eGFR, smoking, drinking, education level, townsend deprivation index, total Metabolic Equivalent Task (MET) minutes per week,coffee intake, tea intake, cereal intake, protein intake, carbohydrate intake, energy intake, total sugars intake, fat intake, comorbidities (history of hypertension, diabetes and dyslipidemia) and fizzy drink intake.

^c^ In Sensitivity analysis IV, the adjustment factors of the mixed-effects Cox regression model included age, sex, race, BMI, eGFR, smoking, drinking, education level, townsend deprivation index, total Metabolic Equivalent Task (MET) minutes per week,coffee intake, tea intake, cereal intake, protein intake, carbohydrate intake, energy intake, total sugars intake, fat intake, comorbidities (history of hypertension, diabetes and dyslipidemia) and UK Biobank assessment center site as random effect.

^d^ In Sensitivity analysis V, the adjustment factors of the competing risk model included age, sex, race, BMI, eGFR, smoking, drinking, education level, townsend deprivation index, total Metabolic Equivalent Task (MET) minutes per week,coffee intake, tea intake, cereal intake, protein intake, carbohydrate intake, energy intake, total sugars intake, fat intake, comorbidities (history of hypertension, diabetes and dyslipidemia).

S**upplemental Table 13. Sensitivity analysis VI-VIII of the association between artificial sweeteners intake and the incidence of CKD by excluding events that occurred within the first two/three/five years of follow-up.**

| **AS intake** | **Sensitivity analysis VI ^a^** | | | **Sensitivity analysis VII ^b^** | | | **Sensitivity analysis VIII ^c^** | | |
| --- | --- | --- | --- | --- | --- | --- | --- | --- | --- |
|  | **Cases/**  **Total** | **IR (95% CI)** | **HR (95% CI);**  P value | **Cases/**  **Total** | **IR (95% CI)** | **HR (95% CI);**  P value | **Cases/**  **Total** | **IR (95% CI)** | **HR (95% CI);**  P value |
| **None-AS** | 4432/  139515 | 2.39 (2.32,2.46) | Ref | 4124/  139207 | 2.22 (2.15,2.29) | Ref | 3405/  138488 | 1.84 (1.78,1.90) | Ref |
| **Low-AS** | 338/  7520 | 3.40 (3.04,3.76) | 1.06 (0.95,1.19) | 319/  7501 | 3.21 (2.86,3.56) | 1.08 (0.96,1.21) | 255/  7437 | 2.57 (2.26,2.89) | 1.04 (0.92,1.19) |
|  |  |  | 0.27 |  |  | 0.19 |  |  | 0.52 |
| **High-AS** | 470/  8183 | 4.37 (3.97,4.76) | 1.21 (1.10,1.34) | 429/  8142 | 3.99 (3.61,4.37) | 1.19 (1.07,1.31) | 352/  8065 | 3.28 (2.94,3.63) | 1.17 (1.04,1.30) |
|  |  |  | <0.001 |  |  | <0.001 |  |  | 0.008 |

Abbreviation: CKD, chronic kidney disease; AS, artificial sweetener; eGFR, estimated glomerular filtration rate; BMI, body-mass index; IR, incidence rate; HR, hazard ratio; CI, confidence interval; Ref, reference.

^a^ Sensitivity analysis VI excluded participants with new-onset CKD events that occurred within the first two years of follow-up.The adjustment factors included age, sex, race, BMI, eGFR, smoking, drinking, education level, Townsend deprivation index, total Metabolic Equivalent Task (MET) minutes per week,coffee intake, tea intake, cereal intake, protein intake, carbohydrate intake, energy intake, total sugars intake, fat intake, comorbidities (history of hypertension, diabetes and dyslipidemia).

^b^ Sensitivity analysis VII excluded participants with new-onset CKD events that occurred within the first three years of follow-up.The adjustment factors included age, sex, race, BMI, eGFR, smoking, drinking, education level, Townsend deprivation index, total Metabolic Equivalent Task (MET) minutes per week,coffee intake, tea intake, cereal intake, protein intake, carbohydrate intake, energy intake, total sugars intake, fat intake, comorbidities (history of hypertension, diabetes and dyslipidemia).

^c^ Sensitivity analysis VIII excluded participants with new-onset CKD events that occurred within the first five years of follow-up.The adjustment factors included age, sex, race, BMI, eGFR, smoking, drinking, education level, Townsend deprivation index, total Metabolic Equivalent Task (MET) minutes per week,coffee intake, tea intake, cereal intake, protein intake, carbohydrate intake, energy intake, total sugars intake, fat intake, comorbidities (history of hypertension, diabetes and dyslipidemia).
